# Double-blind, sham-controlled, pilot study of trigeminal nerve stimulation for autism spectrum disorder

**DOI:** 10.1016/j.neurot.2026.e00838

**Published:** 2026-01-29

**Authors:** Jae Hyun Han, Ye Rim Kim, Yoojeong Lee, Youngmin Park, Dohyoung Kim, Guiyoung Bong, Hee Jeong Yoo

**Affiliations:** a Department of Psychiatry, Seoul National University Bundang Hospital, Seongnam, Republic of Korea; b Nu Eyne Co., Ltd., Seoul, Republic of Korea; c Department of Psychiatry, Seoul National University College of Medicine, Seoul, Republic of Korea

**Keywords:** Autism spectrum disorder, Trigeminal nerve stimulation, Neuromodulation, Randomized controlled trial, Quantitative electroencephalography, Child and adolescent psychiatry

## Abstract

Trigeminal nerve stimulation (TNS) is a minimal-risk, noninvasive neuromodulation method with growing evidence of efficacy across psychiatric conditions. However, its safety and potential effects in autism spectrum disorder (ASD) remain underexplored. This exploratory pilot study aimed primarily to evaluate the safety and tolerability, and secondarily to explore changes in ASD-related symptoms - including impairments in social communication and reciprocity, attention, executive functioning, emotional regulation, sleep, and sensory processing - in children with ASD, and to examine associated changes using quantitative electroencephalography (qEEG). This double-blind, sham-controlled, randomized exploratory pilot trial enrolled 29 children aged 7–12 years with ASD. The participants were randomized to receive 28 nightly sessions of active or sham TNS over 4 weeks. At baseline and week 4, we assessed safety, clinical outcomes and Clinical Global Impression scales, in addition to analyzing qEEG band power. No serious adverse events were observed, and TNS was well tolerated. Exploratory analyses showed nominal between-group differences (unadjusted) favoring the TNS group in maladaptive behavior (Vineland-II: 1.38 vs 0.08; *p* = .017) and social reciprocity (Social Responsiveness Scale-2: 12.07 vs −1.43; *p* = .025). Exploratory qEEG analyses revealed decreased gamma/high-frequency and increased alpha power in the left frontal and parietal regions, changes that significantly correlated with improvements in social (*r* = −0.917; *p* = .001) and overall (*r* = −0.680; *p* = .030) functioning. TNS was safe and showed preliminary evidence of potential benefits in improving behavioral and social functioning in children with ASD. Larger trials are required to confirm these findings. Clinical trial registration information: http://clinicaltrials.gov/; NCT06233279.

## Introduction

Autism spectrum disorder (ASD) is a neurodevelopmental disorder defined by social communication impairments and restricted, repetitive behaviors, with severity ranging from mild to severe [1,2]. Individuals with ASD experience developmental challenges including maladaptive behavior, sleep disturbances, deficits in attention and executive functioning, and emotional difficulties [[3], [4], [5], [6], [7]]. These impairments persist throughout development, limiting learning and social participation [[8], [9], [10]]. Social deficits and maladaptive behaviors particularly impact development and increase caregiver stress [11]. Atypical antipsychotics have shown efficacy in reducing irritability and behavioral outbursts in ASD [12], but do not address core social communication deficits [13,14]. Side effects and parental concerns limit medication adherence [15]. In addition, several recent pharmacological agents have been investigated for targeting core ASD symptoms, including oxytocin [16], balovaptan [17], arbaclofen [18], and bumetanide [19]. However, these trials have not demonstrated consistent efficacy and were often associated with adverse effects, thereby limiting their clinical applicability.

Behavioral interventions like Applied Behavior Analysis and Naturalistic Developmental Behavioral Interventions remain standard care in early ASD treatment [20,21], showing benefits in language and social skills [22]. However, their efficacy remains modest [23], with limited skill generalization and clinical gains [24]. These interventions require 20–40 h weekly, burdening families and care systems [[25], [26], [27]]. Given these limitations, interest has grown in complementary interventions [25,28].

Trigeminal nerve stimulation (TNS) has shown efficacy in modulating attention and emotional regulation in ADHD and depression [29], with trials reporting improved executive functioning and prefrontal network activation [30,31]. Studies show TNS activates brain regions involved in attention control that overlap with ASD neural substrates [32]. Given that ASD involves deficits in social communication, attention, emotional regulation, and executive function, TNS may engage shared neural circuits for similar benefits [29]. In addition, while conventional vagus nerve stimulation (VNS) has been investigated as a neuromodulatory intervention in epilepsy and psychiatric disorders, including exploratory use in children with ASD comorbid with epilepsy, its applicability is limited due to the need for surgical implantation and associated risks [33]. At the same time, it is noteworthy that less invasive approaches such as transcutaneous auricular VNS have also been explored, indicating that not all forms of VNS necessarily require surgery [34]. In contrast, TNS is a fully noninvasive approach, delivered through external electrodes, and has demonstrated favorable tolerability in pediatric populations [30,31]. These advantages make TNS particularly feasible for children with ASD, offering a safer and more accessible alternative to invasive neuromodulation strategies. The trigeminal nerve projects to brainstem structures like the locus coeruleus and nucleus tractus solitarius, which are key to the potential therapeutic effects of TNS on the arousal and attentional systems implicated in ASD [35]. TNS is well-tolerated in pediatric populations, with minimal adverse events (AE) in ADHD trials, suggesting feasibility for children with ASD [30,31]. These neurophysiological, neuroimaging, and clinical findings support TNS as a safe, noninvasive neuromodulatory intervention for ASD deficits. Resting-state quantitative EEG (qEEG) studies in ASD have consistently demonstrated atypical spectral patterns, often described as a “U-shaped” profile with elevated low-frequency (delta, theta) and high-frequency (beta, gamma) activity, accompanied by reduced alpha power. These abnormalities are thought to reflect atypical cortical excitability and disrupted functional connectivity. Neuromodulatory interventions such as TNS may normalize these pathological EEG features. However, to our knowledge, no published studies have evaluated TNS in ASD. Therefore, this pilot study evaluated TNS as an intervention for children with ASD using a double-blind, sham-controlled design. The primary objective was to assess TNS safety and tolerability in pediatric patients with ASD. Secondary outcomes explored TNS-related changes in functioning, core autism symptoms, executive functioning, sleep, anxiety, sensory processing, and clinical improvement. Quantitative electroencephalography (qEEG) examined neurophysiological changes associated with ASD symptoms and their correlation with clinical improvements. We hypothesized that nightly TNS would reduce delta, beta, and gamma power while enhancing alpha activity, particularly in frontal and parietal regions, and that such neurophysiological changes would be associated with improvements in social communication and adaptive functioning.

## Materials and methods

### Study design

This was a 4-week, double-blind, sham-controlled trial with four outpatient visits: screening, baseline, and follow-up visits at weeks 2 and 4. Remote assessments were conducted at weeks 1 and 3. A child psychiatrist performed screening procedures, confirming ASD diagnosis based on DSM-5 criteria [1], Korean version of the Autism Diagnostic Observation Schedule, Second edition (K-ADOS-2) [36], and Korean version of the Autism Diagnostic Interview-Revised (K-ADI-R) [37], while verifying inclusion and exclusion criteria. Medical eligibility was determined through vital signs, laboratory tests, ECG, qEEG, waking EEG, and physical examinations. Adverse events (AEs) were reviewed at screening and monitored weekly through week 4. Efficacy was assessed using the Clinical Global Impression and standardized measures across multiple domains: cognitive and adaptive functioning (K-WISC-IV, K-Vineland-II), core autism symptoms and social reciprocity (ABC-II, AQ, SRS-2, CARS-2, SCQ), executive function and attention (CCTT, Stroop, ATA, WCST, K-ARS), sleep (K-CSHQ), anxiety (K-CBCL, K-STAIC), and sensory processing (SSP-2), administered at baseline, week 2, and week 4. At baseline, caregivers received training in electrode placement, mobile application usage, and device operation. Participants received the investigational device and logbook for recording nightly usage. Participants were randomly assigned in a 1:1 ratio to either the intervention or the control group. An independent statistician, who was not involved in participant enrollment, generated the random allocation sequence using the proc plan procedure in version 9.4 (SAS Institute Inc., Cary, NC, USA). A block randomization design with randomly varying block sizes was employed to ensure balance between the groups throughout the trial. Allocation concealment was maintained by restricting access to the randomization list exclusively to the statistician until interventions were assigned. Participants, caregivers, investigators, outcome assessors, and data analysts were blinded to group allocation until the study database was locked. Active or sham TNS were applied nightly for 8 h during sleep, with weekly behavioral assessments. Blinding was maintained by providing active and sham devices that were identical in appearance, weight, and operation. After 4 weeks, qEEG and waking EEG were repeated, and device use ended.

### Participants

Participants were recruited through outpatient advertisements at the Department of Psychiatry, Seoul National University Bundang Hospital, and Internet community forums between November 2022 and April 2024. Follow-up continued until the end of the study, with additional monitoring for participants who experienced adverse events until symptom resolution. Children aged 7–12 years diagnosed with ASD based on the K-ADOS-2, K-ADI-R, and clinical judgment of a board-certified child psychiatrist using DSM-5 criteria were included. Inclusion criteria were: full-scale IQ score ≥70 on the Korean Wechsler Intelligence Scale for Children, Fourth Edition (K-WISC-IV) [38]; adequate verbal communication; maintenance of prescribed medications without changes throughout the study; and ability to cooperate with study procedures, including EEG. Permitted medications included methylphenidate, atomoxetine, aripiprazole, risperidone, and selective serotonin reuptake inhibitors like escitalopram, sertraline, fluoxetine, and paroxetine. Exclusion criteria included clinically significant behavioral or emotional regulation issues (e.g., severe aggression, persistent self-injury, or uncontrollable tantrums) that could interfere with the treatment process, the presence of psychotic symptoms, risk of self-harm or harm to others, acute or chronic severe medical or psychiatric conditions, a history of seizures, or any other conditions deemed prohibitive for the investigational medical device. Children requiring sleep medications were excluded to minimize confounding influences on sleep-related outcomes and EEG measures. Parents and children received study explanations and provided written informed consent. The study was approved by the Institutional Review Board of Seoul National University Bundang Hospital (IRB No. 2206-762-001). Based on previous pilot TNS studies in similar pediatric neurodevelopmental populations that enrolled approximately 30 participants [30], the sample size for this study was determined primarily on the basis of feasibility rather than a formal power calculation. Allowing for an anticipated dropout rate of 20%, the target enrollment was 15 participants per group, for a total of 30 participants.

### TNS intervention

The TNS intervention was based on previous research on depression [39,40], post-traumatic stress disorder [40], and ADHD [30,31]. Stimulation was delivered using a P01 personal neurostimulator (Nu Eyne Co., Ltd. Korea). Parents placed patch electrodes on the child’s forehead for connection to the rechargeable stimulator unit. Active stimulation used 120 Hz frequency symmetric biphasic pulse with 250 μs pulse width, and 30 s on/off duty cycle. The stimulation waveform consisted of anodic-leading and cathodic-leading biphasic pulses delivered in an alternating sequence. Stimulation intensity was set at 2–4 mA via titration to a tolerable level. The sham device delivered a single symmetric biphasic pulse (0.5 mA, 50 μs) per every 30-s interval for monitoring electrode–skin contact. Because the biphasic pulse parameters used in the sham device were below the somatosensory threshold [41], participants in the control group would not perceive the stimulation. Active and sham devices were identical. Participants applied the device during nighttime sleep after 8:00 p.m. for 8 h (±60 min), daily for four weeks, totaling 28 sessions. Participants were instructed to initiate stimulation at bedtime and maintain it throughout their natural sleep duration until morning awakening. The device was not limited to a fixed 8-h period; rather, stimulation was applied for the entirety of each participant’s actual sleep time, ensuring consistent nighttime exposure regardless of bedtime or total sleep duration. At baseline, families received the device and treatment diary. Parents installed the mobile application and received training from trained research staff on the use of the stimulator and the mobile application. To ensure fidelity, all caregivers received standardized training by research staff on proper electrode placement and device operation. Fidelity was monitored by weekly telephone check-ins and review of device-recorded usage logs via the mobile application. When deviations from protocol were identified, additional guidance and retraining were provided. Caregivers completed daily treatment diaries to support adherence, monitored through regular reviews. For noncompliance (≥30% missed sessions), investigators assessed reasons and could withdraw participants. Diaries were collected at end-of-treatment to assess compliance.

### Outcome measures

The primary outcome was device safety, defined as the proportion of participants experiencing one or more device-related AEs over the 4-week intervention period. Safety was assessed systematically at each weekly visit. Instead of a fixed symptom checklist, investigators conducted structured open-ended inquiries to capture any potential AEs, including psychiatric symptoms, dermatologic reactions, headache, drowsiness, or trigeminal nerve-related complaints. In addition, any AEs spontaneously reported by participants at any time were recorded. Participants with persistent AEs at the final visit were followed until resolution. The secondary outcome was improvement in ASD-related symptoms, assessed using within-group changes and between-group comparisons of scores on efficacy measures. These domains include functioning level, core autism symptoms, executive functioning, sleep, anxiety, sensory processing, and clinical global impression. Functioning was evaluated using the Korean Wechsler Intelligence Scale for Children–Fourth Edition (K-WISC-IV) [38], a standardized measure of intellectual ability, and Korean Vineland Adaptive Behavior Scales, Second Edition (K-Vineland-II) [42], which assesses adaptive functioning in everyday settings. Core ASD symptoms were measured using clinician-administered and parent-reported tools: Aberrant Behavior Checklist-II (K-ABC-II) [43], which evaluates maladaptive behaviors; Korean Autism Spectrum Quotient (AQ) [44], a screening tool for autistic traits; Korean Social Responsiveness Scale–Second Edition (SRS-2) [45], which measures deficits in reciprocal social interaction and communication; Korean Childhood Autism Rating Scale–Second Edition (K-CARS-2) [46], a clinician-rated tool of autism symptom severity; and Korean Social Communication Questionnaire (SCQ) [47], a caregiver-report questionnaire of communication and social functioning. Executive function was assessed using CCTT-1 and CCTT-2 [48], which assess sustained and divided attention and cognitive flexibility; Stroop Color and Word Test [49], which measures attentional shifting and inhibitory control; Advanced Test of Attention (ATA) [50], a computerized task that directly evaluates attentional capacity; Wisconsin Card Sorting Test (WCST) [51], which assesses set-shifting and cognitive flexibility; and Korean ADHD Rating Scale (K-ARS) [52], a caregiver-reported measure of inattentive and hyperactive-impulsive symptoms. Among these measures, attention was directly evaluated with the ATA, and attentional processes were also captured indirectly through the CCTT (sustained and divided attention), Stroop (attentional shifting and inhibition), WCST (attentional flexibility), and the inattention subscale of the K-ARS. Sleep was evaluated using Korean Children’s Sleep Habits Questionnaire (K-CSHQ) [53], a caregiver-report measure of sleep behaviors and problems; anxiety using Korean Child Behavior Checklist (K-CBCL) [54], which captures caregiver-reported internalizing and externalizing symptoms, and Korean State-Trait Anxiety Inventory for Children (K-STAIC) [55], a self-report tool measuring both transient state and trait anxiety; and sensory processing using Short Sensory Profile–2 (SSP-2) [56], a caregiver-report instrument capturing sensory modulation difficulties. Clinical impression was rated using the Clinical Global Impression–Severity (CGI-S), which is a clinician-rated 7-point scale of overall illness severity, and the Clinical Global Impression–Improvement (CGI-I), a 7-point scale of global improvement relative to baseline. These scales are widely used in psychiatric and neurodevelopmental clinical trials as global indices of treatment response [57].

### EEG recording and quantitative analysis

Resting-state EEG data were collected while participants were awake to assess neurophysiological functioning. Participants were seated comfortably in a quiet room and instructed to keep their eyes closed for approximately 5 min to minimize external stimuli and artifacts. EEG signals were recorded using a Comet Plus (Natus Neurology, USA) with electrodes positioned according to the International 10–20 system. The sampling rate was set at 200 Hz, and electrode impedances were maintained below 10 kΩ.

qEEG data were acquired during a 7-min resting-state session with eyes closed using a 64-channel EEG system (Compumedics). Data were referenced between Cz and Cpz, grounded at AFz, and sampled at 1000 Hz with impedance below 5 kΩ. Eye movements were monitored using electrooculographic electrodes. Data processing used EEGLAB in MATLAB, including band-pass filtering (0.1–50 Hz), common average referencing, and independent component analysis (ICA) to remove artifacts. Components were inspected based on topography and spectral features, and non-neural artifacts were excluded. Spectral power analysis used fast Fourier transform on a 5-min segment from min 1 to 6. Relative power was computed across frequency bands: delta (1–4 Hz), theta (4–8 Hz), alpha (8–12 Hz), beta (12–25 Hz), high-beta (25–30 Hz), gamma (30–40 Hz), and high-gamma (40–50 Hz). Between-condition comparisons used Wilcoxon tests, with significance at *p* < .05. Pearson’s correlations were conducted between power changes and efficacy outcomes within the intervention group, excluding outliers with absolute Z-scores > 2.

### Statistical analysis

Statistical analyses used SAS version 9.4 (SAS Institute Inc., Cary, NC, USA). Continuous variables are summarized with descriptive statistics, categorical variables as frequencies and percentages. Independent t-tests compared continuous variables between groups when normality was met; otherwise, Wilcoxon rank-sum tests were used. Paired t-tests or Wilcoxon signed-rank tests analyzed within-group changes. Chi-square or Fisher’s exact tests were used for categorical variables based on cell frequencies. To evaluate treatment effects at the group level, baseline-to-week-4 change scores were calculated for each efficacy outcome, and between-group differences in these change scores were compared using independent t-tests (or Wilcoxon rank-sum tests if non-parametric). This clarified the comparison of treatment response between the active and sham groups. While the primary evaluation of efficacy relied on between-group comparisons, within-group analyses (pre-vs. post-treatment) were conducted solely as secondary, exploratory measures. These analyses were performed to descriptively examine symptom trajectories within each study arm and to obtain preliminary effect size estimates to inform the design and power calculations of future confirmatory trials, and were not used to draw conclusions regarding treatment efficacy. For the exploratory correlational analyses examining associations between changes in qEEG measures and secondary clinical outcomes, the Benjamini–Hochberg procedure was applied to control the False Discovery Rate (FDR) due to the large number of multiple comparisons. For these specific analyses, statistical significance was determined based on FDR-adjusted *p*-values. The primary analysis was on the intention-to-treat (ITT) population, defined as participants who used the device once and had post-baseline efficacy data. Additional per-protocol (PP) analyses excluded those with major deviations or poor adherence (<70% compliance). Safety outcomes included all device users. Missing efficacy data were imputed with the last observation carried forward (LOCF) method. For all efficacy and safety comparisons (excluding the qEEG correlations described above), results were considered statistically significant at a two-sided *p* <.05 without adjustment for multiplicity, consistent with the pilot nature of the study.

## Results

### Baseline demographic characteristics and diagnostic profile

**Fig. 1:**
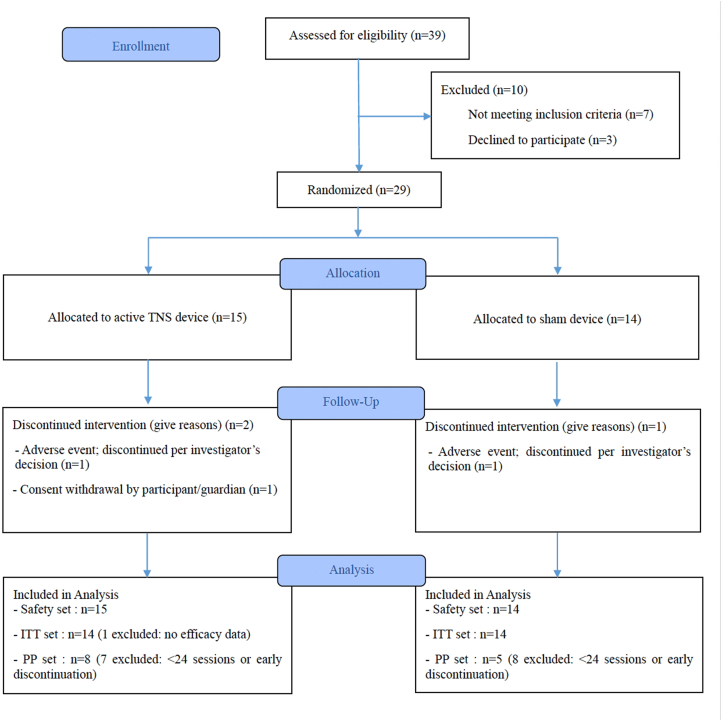
Flow diagram of study participants.

Fig. 1 shows participant flow through the trial. Of 39 children screened, 10 were excluded (7 did not meet criteria, 3 withdrew consent). Twenty-nine participants were randomized to intervention (*n* = 15) or control (*n* = 14) groups. In the intervention group, 13 completed the study; 2 discontinued due to an AE (*n* = 1) or withdrawal (*n* = 1). In the control group, 13 completed, with one AE discontinuation. All randomized participants were included in safety analysis. One participant in the intervention group discontinued the study before completing any post-baseline assessments; therefore, efficacy data were unavailable, and this participant was not included in the ITT analysis. As a result, 28 participants were included in the ITT population (intervention: *n* = 14; control: *n* = 14). For PP analysis, 15 were excluded due to insufficient device usage or early discontinuation, yielding 13 participants (intervention: *n* = 8; control: *n* = 5). Baseline characteristics of the ITT population are shown in Table 1. Groups were well balanced. Mean age was 8.21 (1.10) years, with 78.6% boys. No significant differences existed between groups in sex distribution or measures (mean height, 134.28 (6.21) cm; weight, 31.89 (8.62) kg). All participants met ASD criteria based on K-ADOS-2 and K-ADI-R, with no significant between-group differences in any domains (*p* >.05). All participants communicated verbally. Prior medication use was reported by 82.1% of participants, mainly nervous system agents and antipsychotics. During the study, 85.7% used concomitant medications, with similar distribution across groups (Supplementary Tables 1 and 2).

**Table 1:** Demographic characteristics and distribution of autism spectrum disorder diagnoses (ITT population).

Category	Intervention Group (*N* = 14)	Control Group (*N* = 14)	Total (*N* = 28)	*p*-value
**Age (years)**				0.1584^b^
N	14	14	28	
Mean [SD]	7.93 [1.07]	8.50 [1.09]	8.21 [1.10]	
Median	8.00	8.50	8.00	
Min, Max	7.00, 10.00	7.00, 10.00	7.00, 10.00	
**Sex, N (%)**				0.6483^c^
Male	12 (85.71)	10 (71.43)	22 (78.57)	
Female	2 (14.29)	4 (28.57)	6 (21.43)	
**Height (cm)**				0.9105^a^
N	14	14	28	
Mean [SD]	134.14 [6.59]	134.41 [6.05]	134.28 [6.21]	
Median	132.40	133.05	132.95	
Min, Max	123.80, 148.30	121.50, 144.00	121.50, 148.30	
**Weight (kg)**				0.9268^b^
N	14	14	28	
Mean [SD]	31.55 [8.40]	32.22 [9.14]	31.89 [8.62]	
Median	28.60	28.65	28.60	
Min, Max	23.80, 51.20	22.00, 52.00	22.00, 52.00	
**Diagnosis based on K-ADOS-2 & K-ADI-R, N (%)**				–
Autism spectrum disorder	14 (100.00)	14 (100.00)	28 (100.00)	
Typical development	0 (0.00)	0 (0.00)	0 (0.00)	
Other	0 (0.00)	0 (0.00)	0 (0.00)	
**K-ADOS2**				
**Social affect (points)**				0.6320^b^
N	14	14	28	
Mean [SD]	11.07 [1.44]	10.93 [1.44]	11.00 [1.41]	
Median	11.00	10.00	11.00	
Min, Max	9.00, 14.00	10.00, 15.00	9.00, 15.00	
**Restricted and repetitive behaviors (points)**				0.8978^b^
N	14	14	28	
Mean [SD]	0.57 [0.65]	0.57 [0.76]	0.57 [0.69]	
Median	0.50	0.00	0.00	
Min, Max	0.00, 2.00	0.00, 2.00	0.00, 2.00	
**Total score (points)**				0.6234^b^
N	14	14	28	
Mean [SD]	11.64 [1.55]	11.50 [1.70]	11.57 [1.60]	
Median	11.50	11.00	11.00	
Min, Max	9.00, 14.00	10.00, 15.00	9.00, 15.00	
**ADOS comparison score (points)**				0.3923^b^
N	14	14	28	
Mean [SD]	7.14 [0.66]	6.93 [1.00]	7.04 [0.84]	
Median	7.00	7.00	7.00	
Min, Max	6.00, 8.00	6.00, 9.00	6.00, 9.00	
**K-ADI-R**				
**Presence of Spoken language**				–
Present	14 (100.00)	14 (100.00)	28 (100.00)	
Absent	0 (0.00)	0 (0.00)	0 (0.00)	
**A(Social communication) (points)**				0.7241^a^
N	14	14	28	
Mean [SD]	20.00 [6.03]	20.79 [5.62]	20.39 [5.73]	
Median	20.00	20.50	20.00	
Min, Max	9.00, 27.00	10.00, 29.00	9.00, 29.00	
**B(Communication) (points)**				0.9816^b^
N	14	14	28	
Mean [SD]	16.07 [4.84]	15.64 [5.51]	15.86 [5.10]	
Median	18.50	15.00	17.50	
Min, Max	7.00, 21.00	9.00, 24.00	7.00, 24.00	
**B(Nonverbal communication) (points)**				0.6831^a^
N	9	9	18	
Mean [SD]	9.56 [3.43]	8.89 [3.37]	9.22 [3.32]	
Median	11.00	9.00	10.00	
Min, Max	3.00, 13.00	4.00, 14.00	3.00, 14.00	
**C(Restricted and repetitive behaviors) (points)**				0.2432^b^
N	14	14	28	
Mean [SD]	4.14 [2.35]	5.64 [4.36]	4.89 [3.52]	
Median	3.00	4.00	4.00	
Min, Max	1.00, 9.00	2.00, 20.00	1.00, 20.00	
**D(Onset before 36 months of age) (points)**				0.7557^a^
N	14	14	28	
Mean [SD]	3.21 [1.37]	3.36 [1.01]	3.29 [1.18]	
Median	3.00	3.00	3.00	
Min, Max	1.00, 5.00	2.00, 5.00	1.00, 5.00	
**WISC-IV index scores**				
**Full-scale IQ**				0.4201^b^
N	14	14	28	
Mean [SD]	89.50 [16.41]	89.71 [9.93]	89.61 [13.31]	
Median	82.00	90.50	87.00	
Min, Max	73.00, 123.00	72.00, 102.00	72.00, 123.00	
**Verbal Comprehension index**				0.4636^a^
N	14	14	28	
Mean [SD]	91.36 [12.77]	94.86 [12.12]	93.11 [12.34]	
Median	90.00	98.00	92.00	
Min, Max	74.00, 117.00	70.00, 114.00	70.00, 117.00	
**Perceptual Reasoning index**				0.4858^a^
N	8	5	13	
Mean [SD]	100.88 [18.63]	94.40 [8.65]	98.38 [15.43]	
Median	99.00	96.00	98.00	
Min, Max	78.00, 133.00	80.00, 102.00	78.00, 133.00	
**Working Memory index**				0.9339^a^
N	8	5	13	
Mean [SD]	91.88 [17.37]	92.80 [21.86]	92.23 [18.31]	
Median	92.00	95.00	92.00	
Min, Max	63.00, 120.00	68.00, 114.00	63.00, 120.00	
**Processing Speed index**				0.3517^a^
N	8	5	13	
Mean [SD]	75.38 [9.29]	80.60 [9.66]	77.38 [9.40]	
Median	76.50	79.00	79.00	
Min, Max	59.00, 88.00	68.00, 95.00	59.00, 95.00	

**Note 1)** N (%): Number (percentage); percentages were calculated using the number of participants in the corresponding group as the denominator.

-: Not analyzed.

a Independent two-sample *t*-test.

b Wilcoxon rank sum test.

c Fisher’s exact test.

### Primary outcomes: safety and tolerability

Among the 29 participants in the safety analysis set, 17 AEs were reported in 12 participants (41.4%), with higher incidence in the control group (64.3%) than intervention group (20.0%) (*p* = .015). Device-related AEs (DRAEs) occurred in four participants (13.8%): one in the intervention group (6.7%) and three in the control group (21.4%), with no serious AEs in either group (Table 2, Supplementary Table 3). Most AEs were mild (94.1%) with one moderate event in the control group (Supplementary Table 4). Specifically, the intervention group had one instance of abnormal EEG without clinical symptoms. In the control group, adverse events included skin and subcutaneous tissue disorders (contact dermatitis, *n* = 1; rash, *n* = 1) and behavioral/neurological symptoms (psychomotor hyperactivity, irritability, and motor tics observed in one participant) (Supplementary Tables 3–5). Most events were unrelated (31.0%) or possibly related (27.6%) to the device. Device use was discontinued in five participants, mainly in the control group. Most device-related events resolved spontaneously; however, the moderate contact dermatitis case in the control group led to study discontinuation. Most AEs resolved without sequelae (31.0%), while 6.9% remained at completion. One intervention group participant was lost to follow-up after an AE (Supplementary Table 4). Overall, the data indicate an acceptable safety profile, with no serious adverse events reported, despite the higher incidence of mild events in the control group.

**Table 2:** Incidence of adverse events as the primary efficacy outcome (Safety population).

Category	Intervention Group (*N* = 15)		Control Group (*N* = 14)		Total (*N* = 29)	
	N (%)	E	N (%)	E	N (%)	E
Adverse events	3 (20.00)	3	9 (64.29)	14	12 (41.38)	17
95 % confidence interval	[4.33, 48.09]		[35.14, 87.24]		[23.52, 61.06]	
*p*-value	0.0155^a^					
Device-related adverse events	1 (6.67)	1	3 (21.43)	5	4 (13.79)	6
95 % confidence interval	[0.17, 31.95]		[4.66, 50.80]		[3.89, 31.66]	
*p*-value	0.3295^b^					
Serious adverse events	0 (0.00)	0	0 (0.00)	0	0 (0.00)	0
95 % confidence interval	[0.00, 21.80]		[0.00, 23.16]		[0.00, 11.94]	
*p*-value	–					
Serious device-related adverse events	0 (0.00)	0	0 (0.00)	0	0 (0.00)	0
95 % confidence interval	[0.00, 21.80]		[0.00, 23.16]		[0.00, 11.94]	
*p*-value	–					

-: Not analyzed.

a. N (%): Number (percentage); percentages were calculated based on participants in each group; E: number of events.

b. For participants with ≥2 adverse events, the individual was counted only once.

a Chi-square test.

b Fisher’s exact test.

### Secondary outcomes: ASD-related symptoms

**Fig. 2:**
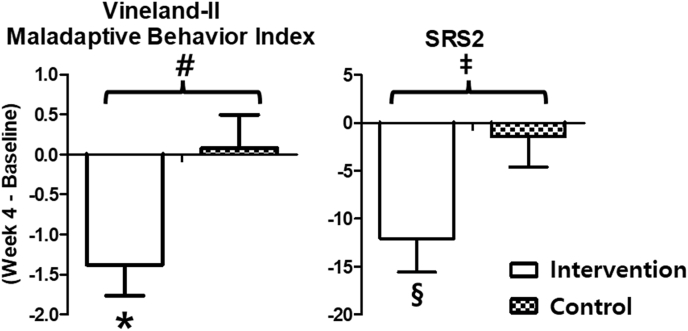
**Changes in Maladaptive Behavior and Social Reciprocity at Week 4.** Mean changes from baseline to week 4 in the Vineland-II Maladaptive Behavior Index and Social Responsiveness Scale–Second Edition (SRS-2) scores in the intervention and control groups. Greater reductions were observed in the intervention group than that in the control group for both maladaptive behaviors (Vineland-II) and social reciprocity (SRS-2). Error bars indicate standard deviation. ∗*p* < .05, within-group comparison (intervention group). #*p* < .05, between-group comparison (Vineland-II). ‡*p* < .05, between-group comparison (SRS-2). §*p* < .05, within-group comparison (intervention group, SRS-2).

Group differences were observed in outcome measures, including maladaptive behavior and social reciprocity. Children in the intervention group showed greater reductions in maladaptive behavior (Vineland Maladaptive Behavior Index: 1.38 [1.39] vs 0.08 [1.44]; *p* = .017) and social reciprocity improvement (SRS-2: 12.07 [12.65] vs −1.43 [10.96]; *p* = .025) than the sham group (Fig. 2). These outcomes are summarized in Supplementary Table 6 and Supplementary Table 7, which present measures of adaptive functioning and social-communication behavior. However, processing speed was slower in the intervention group (CCTT-1: 26.43 [10.26] vs. 18.64 [4.65] s; *p* = .019) (Supplementary Table 8). Sleep habits improved more in the intervention group at week 2 (K-CSHQ: 0.93 [4.29] vs. 1.21 [1.93]; *p* = .048) (Supplementary Table 10). Within-group analyses showed improvements from baseline to week 4 in the intervention group in Vineland Socialization Index (4.54 [5.65], *p* = .013), Composite Standard Score (7.00 [8.97], *p* = .016), and Adaptive Behavior Composite score (2.23 [2.86], *p* = .016) (Supplementary Table 6). The intervention group showed improvements in ABC-II at week 2 (5.43 [9.25], *p* = .047) (Supplementary Table 11), SCQ scores at week 4 (−3.43 [4.09], *p* = .003) (Supplementary Table 12), CCTT-2 times (−15.00 [19.78], *p* = .004) (Supplementary Table 8), and Stroop test domains (Supplementary Table 9). The intervention group showed reductions in K-ARS scores (*p* = .004) (Supplementary Table 13), K-CBCL behaviors (*p* = .004) (Supplementary Table 14), and SSP-2 sensory seeking (*p* = .039) (Supplementary Table 15). Overall, the intervention group showed greater improvements in maladaptive behaviors, social reciprocity, and sleep disturbance.

### Quantitative EEG findings

**Fig. 3:**
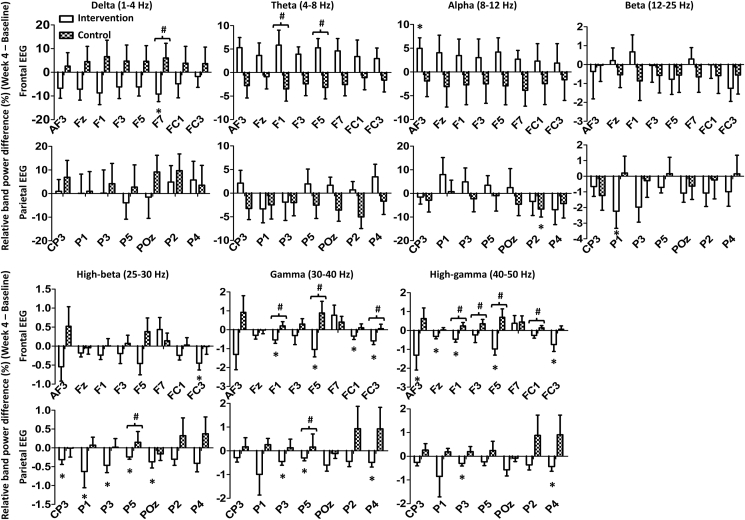
**Relative band power differences in frontal and parietal EEG regions.** Relative band power differences between Week 4 visit and Baseline visit (Mean ± SEM). The upper line shows the relative band power difference of the left frontal EEG (AF3, Fz, F1, F3, F5, F7, FC1, and FC3), and the lower line shows the relative band power difference of the parietal EEG (CP3, P1, P3, P5, POz, P2, P4). Frontal and parietal EEG electrodes that showed significant relative band power differences between or within the groups were selected. #Wilcoxon rank-sum test (*p* < .05); ∗ Wilcoxon signed-rank test (*p* < .05).

Among the intent-to-treat population, 22 participants (10 intervention, 12 control) completed qEEG assessments at baseline and week 4. Within-group comparisons showed significant changes in relative band power for the intervention group in left frontal and parietal regions (Fig. 3; Supplementary Table 16, Supplementary Table 17). Decreases occurred in delta power (F7), high-beta power (FC3), gamma power (F1, F5, FC1, FC3), and high-gamma power (AF3, Fz, F1, F5, FC3), while alpha power increased at AF3. In the left parietal region, high-frequency power decreased significantly at P1, P3, and P5. Power reductions were also observed at POz, P2, and P4. The control group showed no significant changes, except increased alpha power at P2 (*p* = .0269). Between-group comparisons of the changes in relative band power from baseline to week 4 revealed significant differences across frequencies and electrode sites (Fig. 3). The intervention group showed greater reductions in delta (F7), gamma (F1, F5, FC3), and high-gamma (F1, F3, F5, FC1) power and increased theta power (F1, F5) versus the control group. At P5, high-beta and gamma powers were significantly lower in the intervention group.

### Correlation analysis with efficacy outcomes

**Fig. 4:**
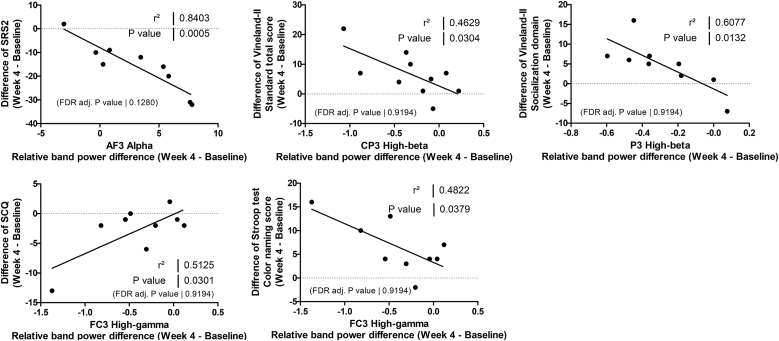
**Significant linear correlations between efficacy outcomes and relative band power difference in the intervention group.** In the intervention group, improvements in SRS-2 scores had a significant linear correlation with increases in left frontal (AF3) alpha relative band power. Improvements in Vineland-II standard total scores and socialization domain scores showed significant linear correlation with decreases in left parietal (CP3, P3) high-beta relative band power. Additionally, Improvements in SCQ scores and Color naming scores on the Stroop test had a significant linear correlation with decreases in left frontal (FC3) high-gamma relative band power (Pearson’s linear correlation, *p* < .05).

Correlation analyses examined whether the direction of qEEG change (increase or decrease) predicted clinical improvement. In uncorrected exploratory analyses, changes of four qEEG relative band powers exhibited nominally significant linear correlation with clinical improvements. Specifically, relative increases in alpha power at AF3 correlated with reductions in SRS-2 scores (*r*^2^ = 0.8403, unadjusted *p* = .0005, FDR-adjusted *p* = .1280). Conversely, greater reductions in high-beta power (CP3, P3) correlated with improvements in Vineland-II scores (CP3 – standard total score: *r*^2^ = 0.4629, unadjusted *p* = .0304, FDR-adjusted *p* = .9194; P3 - social domain: *r*^2^ = 0.6077, unadjusted *p* = .0132, FDR-adjusted *p* = .9194). Similarly, greater reductions in high-gamma (FC3) also correlated with improvements in SCQ (*r*^2^ = 0.5125, unadjusted *p* = .0301, FDR-adjusted *p* = .9194) and Color naming scores of Stroop test (*r*^2^ = 0.4822, unadjusted *p* = .0379, FDR-adjusted *P* = .9194) (Fig. 4).

## Discussion

This randomized, controlled pilot study evaluated the safety and preliminary efficacy of TNS for children with ASD. The primary outcome was safety, with AEs monitored during the 4-week intervention. AE incidence was higher in the control group than that in the active TNS group despite no active stimulation. This observation may be attributed to heightened awareness of minor symptoms or physical discomfort from the sham device in a clinical trial setting. While expectancy-related factors (e.g., nocebo-like effects) are theoretically possible, the modest magnitude of between-group differences in efficacy outcomes suggests that expectation-disconfirmation is unlikely to be the primary driver of the increased AE reporting in the control group. No serious AEs or DRAEs occurred in either group; most AEs were mild and resolved without sequelae. These findings support TNS device safety in pediatric ASD patients.

Significant between-group differences were observed in key domains. The TNS group showed greater improvements in maladaptive behavior (Vineland Maladaptive Behavior Index), social reciprocity (SRS-2), and sleep-related functioning (K-CSHQ) compared to controls. These results suggest TNS may positively affect socioemotional and behavioral regulation. The SRS-2 total score showed significant improvement in the TNS group. Reductions in maladaptive behaviors may have contributed to enhanced social engagement. Given the correlation between behavioral regulation and social functioning in children with ASD, improvements in self-regulatory capacity may manifest as reduced externalizing behaviors and increased social engagement. The observed SRS improvements may reflect effects of reduced maladaptive behavior through TNS-induced behavioral control. However, this interpretation requires further investigation. TNS group participants required longer time to complete CCTT-1, possibly reflecting cognitive burden or reduced processing speed. Further research is needed to determine if this represents a cognitive side effect.

Although the effect sizes of the between-group differences were modest, they may represent clinically meaningful signals given the short 4-week intervention period. Furthermore, because these improvements were captured by parent-rated measures (e.g., SRS-2, SCQ, Vineland-II), they suggest that TNS-related neurophysiological changes may be accompanied by observable benefits in daily social functioning. However, it is important to note that while these statistical improvements are promising, they should be interpreted as preliminary signals rather than definitive evidence of real-world clinical benefit. Future adequately powered trials utilizing pre-specified responder criteria and broader functional outcomes are necessary to formally establish the clinical meaningfulness of these findings. Taken together with the favorable safety profile, these findings support the potential role of TNS as a safe adjunctive intervention, while underscoring the need for confirmation in larger trials.

Within-group improvements were observed in the TNS group across adaptive behavior (Vineland-II), executive function (CCTT-2, Stroop), social communication (SCQ), and ADHD symptoms (K-ARS). While between-group differences did not reach statistical significance for all measures, the TNS group exhibited consistent within-group improvements across adaptive, executive, and attentional domains, suggesting a potential trend toward developmental benefits that warrants further validation in larger samples. These within-group analyses were conducted primarily to estimate effect sizes and describe symptom trajectories to inform the design of future confirmatory trials. Resting-state EEG power in patients with ASD shows a U-shaped profile with excessive power in low- and high-frequency bands, potentially related to GABAergic interneuron dysfunction. Studies report increased power in left frontal and parietal regions, with elevated gamma power possibly reflecting local overconnectivity [58].

In our qEEG analysis, significant changes in relative band power were observed at left frontal and parietal electrodes after 4 weeks of TNS. These changes normalized the pathological U-shaped profile, with reductions in delta, beta, high-beta, gamma, and high-gamma powers and increases in theta and alpha powers. Regarding the relationship between these neurophysiological changes and clinical improvements, we applied false discovery rate (FDR) adjustment to account for multiple comparisons. Although the correlations did not retain statistical significance after FDR adjustment, the direction of associations remained consistent. Specifically, increases in alpha power and decreases in high-gamma power at left frontal sites showed trends of association with improvements in social functioning, while reductions in high-beta power at left parietal sites were observed to track with improved functioning. These findings, while preliminary, suggest potential neuromodulatory effects of TNS requiring further investigation. This study had key strengths: participants using psychotropic medications were included, better reflecting real-world clinical populations where TNS is likely to be applied. By controlling for medication effects, we evaluated TNS in an ecologically valid manner. Additionally, we used validated assessment tools spanning behavioral symptoms, social functioning, executive function, sensory processing, and neurophysiological markers, enabling holistic evaluation of treatment effects across ASD domains.

This study has limitations. First, the sample size was small due to the pilot design and was determined based on feasibility and precedents in similar pilot studies rather than a formal power calculation, limiting statistical power. Second, although a sham control group was used, blinding success was not formally assessed, affecting subjective outcome interpretation. Third, no long-term follow-up was conducted, limiting evaluation of treatment durability. Fourth, while FDR adjustment was applied to exploratory qEEG correlations, strictly confirmatory correction methods were not applied to secondary efficacy outcomes to avoid inflating Type II error in this pilot phase, potentially increasing false-positive results. Fifth, the absence of a cross-over design limited the ability to control for inter-individual variability in treatment response. Finally, this single-site study with a homogeneous population limits generalizability. Future multicenter trials with larger samples, longer follow-up, and objective measures are needed to extend these findings.

This randomized controlled pilot trial shows TNS is safe and well-tolerated for children with ASD, highlighting potential benefits in adaptive functioning, social reciprocity, and symptom regulation. While limited by sample size and duration, the results justify further large-scale trials to establish clinical efficacy in pediatric ASD populations.

## Author contributions

Hee Jeong Yoo had full access to all of the data in the study and takes responsibility for the integrity of the data and the accuracy of the data analysis.

Concept and design: Jae Hyun Han, Hee Jeong Yoo, Dohyoung Kim.

Acquisition, analysis, or interpretation of data: All authors.

Drafting of the manuscript: Jae Hyun Han.

Critical review of the manuscript for important intellectual content: All authors.

Statistical analysis: Jae Hyun Han, Youngmin Park.

Obtained funding: Dohyoung Kim.

Administrative, technical, or material support: Ye Rim Kim, Yoojeong Lee, Guiyoung Bong.

Supervision: Hee Jeong Yoo.

## Declaration of competing interest

The authors declare the following financial interests/personal relationships which may be considered as potential competing interests: Hee Jeong Yoo reports financial support was provided by Nu Eyne Co., Ltd. Hee Jeong Yoo reports a relationship with Nu Eyne Co., Ltd. that includes: funding grants. The other authors declare that they have no known competing financial interests or personal relationships that could have appeared to influence the work reported in this paper.
