# Supplementary material for: Double-blind, sham-controlled, pilot study of trigeminal nerve stimulation for autism spectrum disorder
Source: Neurotherapeutics. 2026 Jan 29;23(1):e00838. doi: 10.1016/j.neurot.2026.e00838 (PMC12976484; doi:10.1016/j.neurot.2026.e00838)
Supplement: Multimedia component 1 [file mmc1.docx]

**Supplementary Table 1. Use of Prior Medications in the ITT Population**

| **Anatomical**  **Therapeutic** | **Intervention Group (N=14)** | | **Control Group (N=14)** | | **Total (N=28)** | |
| --- | --- | --- | --- | --- | --- | --- |
|  | **N (%)** | **E** | **N (%)** | **E** | **N (%)** | **E** |
| Total | 11 (78.57) | 40 | 12 (85.71) | 32 | 23 (82.14) | 72 |
| p-value | 1.0000 | | | |  |  |
| **NERVOUS SYSTEM** | **9 (64.29)** | **26** | **10 (71.43)** | **22** | **19 (67.86)** | **48** |
| PSYCHOANALEPTICS | 9 (64.29) | 14 | 9 (64.29) | 15 | 18 (64.29) | 29 |
| PSYCHOLEPTICS | 7 (50.00) | 11 | 6 (42.86) | 7 | 13 (46.43) | 18 |
| ANALGESICS | 1 (7.14) | 1 | 0 (0.00) | 0 | 1 (3.57) | 1 |
| **RESPIRATORY SYSTEM** | **3 (21.43)** | **10** | **4 (28.57)** | **7** | **7 (25.00)** | **17** |
| ANTIHISTAMINES FOR SYSTEMIC USE | 1 (7.14) | 1 | 3 (21.43) | 3 | 4 (14.29) | 4 |
| COUGH AND COLD PREPARATIONS | 2 (14.29) | 4 | 1 (7.14) | 1 | 3 (10.71) | 5 |
| DRUGS FOR OBSTRUCTIVE AIRWAY  DISEASES | 2 (14.29) | 2 | 1 (7.14) | 1 | 3 (10.71) | 3 |
| NASAL PREPARATIONS | 2 (14.29) | 3 | 1 (7.14) | 2 | 3 (10.71) | 5 |
| **SENSORY ORGANS** | **0 (0.00)** | **0** | **2 (14.29)** | **2** | **2 (7.14)** | **2** |
| OPHTHALMOLOGICALS | 0 (0.00) | 0 | 2 (14.29) | 2 | 2 (7.14) | 2 |
| **ALIMENTARY TRACT AND METABOLISM** | **1 (7.14)** | **3** | **0 (0.00)** | **0** | **1 (3.57)** | **3** |
| ANTIDIARRHEALS, INTESTINAL  ANTIINFLAMMATORY/ANTIINFECTIVE AGENTS | 1 (7.14) | 1 | 0 (0.00) | 0 | 1 (3.57) | 1 |
| DRUGS FOR ACID RELATED DISORDERS | 1 (7.14) | 1 | 0 (0.00) | 0 | 1 (3.57) | 1 |
| DRUGS FOR FUNCTIONAL  GASTROINTESTINAL DISORDERS | 1 (7.14) | 1 | 0 (0.00) | 0 | 1 (3.57) | 1 |
| **ANTINEOPLASTIC AND**  **IMMUNOMODULATING AGENTS** | **0 (0.00)** | **0** | **1 (7.14)** | **1** | **1 (3.57)** | **1** |
| ENDOCRINE THERAPY | 0 (0.00) | 0 | 1 (7.14) | 1 | 1 (3.57) | 1 |
| **CARDIOVASCULAR SYSTEM** | **1 (7.14)** | **1** | **0 (0.00)** | **0** | **1 (3.57)** | **1** |
| BETA BLOCKING AGENTS | 1 (7.14) | 1 | 0 (0.00) | 0 | 1 (3.57) | 1 |

**a.** N (%): Number (percentage); calculated based on participants in each group; E: number of events
**b.** Participants with ≥2 prior medications were counted more than once.
**c.** Medications coded using the Anatomical and Therapeutic levels of the WHO ATC classification (2024).

^$^: Chi-square test

**Supplementary Table 2. Use of concomitant medications in the ITT population**

| **Anatomical**  **Therapeutic** | **Intervention**  **Group (N=14)** | | **Control**  **Group (N=14)** | | **Total (N=28)** | |
| --- | --- | --- | --- | --- | --- | --- |
|  | **N (%)** | **E** | **N (%)** | **E** | **N (%)** | **E** |
| Total | 11 (78.57) | 53 | 13 (92.86) | 66 | 24 (85.71) | 119 |
| p-value | 0.5956^&^ | | | |  |  |
| **NERVOUS SYSTEM** | **10 (71.43)** | **25** | **10 (71.43)** | **22** | **20 (71.43)** | **47** |
| PSYCHOANALEPTICS | 9 (64.29) | 12 | 9 (64.29) | 14 | 18 (64.29) | 26 |
| PSYCHOLEPTICS | 7 (50.00) | 10 | 5 (35.71) | 6 | 12 (42.86) | 16 |
| ANALGESICS | 3 (21.43) | 3 | 2 (14.29) | 2 | 5 (17.86) | 5 |
| **RESPIRATORY SYSTEM** | **4 (28.57)** | **14** | **8 (57.14)** | **23** | **12 (42.86)** | **37** |
| COUGH AND COLD PREPARATIONS | 4 (28.57) | 6 | 5 (35.71) | 6 | 9 (32.14) | 12 |
| ANTIHISTAMINES FOR SYSTEMIC USE | 2 (14.29) | 2 | 6 (42.86) | 7 | 8 (28.57) | 9 |
| NASAL PREPARATIONS | 2 (14.29) | 3 | 5 (35.71) | 6 | 7 (25.00) | 9 |
| DRUGS FOR OBSTRUCTIVE AIRWAY DISEASES | 2 (14.29) | 2 | 3 (21.43) | 3 | 5 (17.86) | 5 |
| OTHER RESPIRATORY SYSTEM  PRODUCTS | 1 (7.14) | 1 | 1 (7.14) | 1 | 2 (7.14) | 2 |
| **ALIMENTARY TRACT AND METABOLISM** | **2 (14.29)** | **8** | **3 (21.43)** | **5** | **5 (17.86)** | **13** |
| DRUGS FOR FUNCTIONAL  GASTROINTESTINAL DISORDERS | 2 (14.29) | 4 | 2 (14.29) | 2 | 4 (14.29) | 6 |
| ANTIDIARRHEALS, INTESTINAL  ANTIINFLAMMATORY/ANTIINFECTIVE AGENTS | 1 (7.14) | 2 | 2 (14.29) | 2 | 3 (10.71) | 4 |
| DRUGS FOR ACID RELATED  DISORDERS | 1 (7.14) | 1 | 1 (7.14) | 1 | 2 (7.14) | 2 |
| DIGESTIVES, INCL. ENZYMES | 1 (7.14) | 1 | 0 (0.00) | 0 | 1 (3.57) | 1 |
| **ANTIINFECTIVES FOR SYSTEMIC USE** | **2 (14.29)** | **3** | **2 (14.29)** | **2** | **4 (14.29)** | **5** |
| ANTIVIRALS FOR SYSTEMIC USE | 2 (14.29) | 2 | 1 (7.14) | 1 | 3 (10.71) | 3 |
| ANTIBACTERIALS FOR SYSTEMIC USE | 1 (7.14) | 1 | 1 (7.14) | 1 | 2 (7.14) | 2 |
| **MUSCULO-SKELETAL SYSTEM** | **1 (7.14)** | **2** | **2 (14.29)** | **3** | **3 (10.71)** | **5** |
| ANTIINFLAMMATORY AND  ANTIRHEUMATIC PRODUCTS | 1 (7.14) | 1 | 1 (7.14) | 2 | 2 (7.14) | 3 |
| OTHER DRUGS FOR DISORDERS OF THE MUSCULO-SKELETAL SYSTEM | 1 (7.14) | 1 | 1 (7.14) | 1 | 2 (7.14) | 2 |
| **SYSTEMIC HORMONAL PREPARATIONS, EXCL. SEX HORMONES AND INSULINS** | **0 (0.00)** | **0** | **3 (21.43)** | **3** | **3 (10.71)** | **3** |
| CORTICOSTEROIDS FOR SYSTEMIC USE | 0 (0.00) | 0 | 3 (21.43) | 3 | 3 (10.71) | 3 |
| **DERMATOLOGICALS** | **0 (0.00)** | **0** | **2 (14.29)** | **2** | **2 (7.14)** | **2** |
| ANTIBIOTICS AND  CHEMOTHERAPEUTICS FOR  DERMATOLOGICAL USE | 0 (0.00) | 0 | 1 (7.14) | 1 | 1 (3.57) | 1 |
| CORTICOSTEROIDS, DERMATOLOGICAL PREPARATIONS | 0 (0.00) | 0 | 1 (7.14) | 1 | 1 (3.57) | 1 |
| **SENSORY ORGANS** | **0 (0.00)** | **0** | **2 (14.29)** | **3** | **2 (7.14)** | **3** |
| OPHTHALMOLOGICALS | 0 (0.00) | 0 | 2 (14.29) | 3 | 2 (7.14) | 3 |
| **ANTINEOPLASTIC AND**  **IMMUNOMODULATING AGENTS** | **0 (0.00)** | **0** | **1 (7.14)** | **1** | **1 (3.57)** | **1** |
| ENDOCRINE THERAPY | 0 (0.00) | 0 | 1 (7.14) | 1 | 1 (3.57) | 1 |
| **CARDIOVASCULAR SYSTEM** | **1 (7.14)** | **1** | **0 (0.00)** | **0** | **1 (3.57)** | **1** |
| BETA BLOCKING AGENTS | 1 (7.14) | 1 | 0 (0.00) | 0 | 1 (3.57) | 1 |
| **[UNKNOWN]** | **0 (0.00)** | **0** | **1 (7.14)** | **2** | **1 (3.57)** | **2** |
| [UNKNOWN] * | 0 (0.00) | 0 | 1 (7.14) | 2 | 1 (3.57) | 2 |

**Supplementary Table 3. Summary of Adverse Events (Safety Population)**

| **Category** | **Intervention Group**  **(N=15)** | | **Control Group**  **(N=14)** | | **Total**  **(N=29)** | |
| --- | --- | --- | --- | --- | --- | --- |
|  | **N (%)** | **E** | **N (%)** | **E** | **N (%)** | **E** |
| **Adverse Events** | 3 (20.00) | 3 | 9 (64.29) | 14 | 12 (41.38) | 17 |
| **Serious Adverse Events** | 0 (0.00) | 0 | 0 (0.00) | 0 | 0 (0.00) | 0 |
| **Severity** |  |  |  |  |  |  |
| Mild | 3 (20.00) | 3 | 8 (57.14) | 13 | 11 (37.93) | 16 |
| Moderate | 0 (0.00) | 0 | 1 (7.14) | 1 | 1 (3.45) | 1 |
| Severe | 0 (0.00) | 0 | 0 (0.00) | 0 | 0 (0.00) | 0 |
| **Causality** |  |  |  |  |  |  |
| Definitely related | 0 (0.00) | 0 | 0 (0.00) | 0 | 0 (0.00) | 0 |
| Probably related | 0 (0.00) | 0 | 2 (14.29) | 2 | 2 (6.90) | 2 |
| Possibly related | 1 (6.67) | 1 | 1 (7.14) | 3 | 2 (6.90) | 4 |
| Unlikely related | 0 (0.00) | 0 | 0 (0.00) | 0 | 0 (0.00) | 0 |
| Not related | 2 (13.33) | 2 | 7 (50.00) | 9 | 9 (31.03) | 11 |
| Not assessable | 0 (0.00) | 0 | 0 (0.00) | 0 | 0 (0.00) | 0 |
| **Actions Taken Regarding Investigational Medical Device** |  |  |  |  |  |  |
| No action taken | 2 (13.33) | 2 | 7 (50.00) | 9 | 9 (31.03) | 11 |
| Intensity reduced | 0 (0.00) | 0 | 0 (0.00) | 0 | 0 (0.00) | 0 |
| Duration reduced | 0 (0.00) | 0 | 0 (0.00) | 0 | 0 (0.00) | 0 |
| Intensity and duration reduced | 0 (0.00) | 0 | 0 (0.00) | 0 | 0 (0.00) | 0 |
| Temporarily discontinued | 1 (6.67) | 1 | 2 (14.29) | 4 | 3 (10.34) | 5 |
| Permanently discontinued | 0 (0.00) | 0 | 1 (7.14) | 1 | 1 (3.45) | 1 |
| **Treatment Other Than Investigational Medical Device** |  |  |  |  |  |  |
| None | 1 (6.67) | 1 | 1 (7.14) | 3 | 2 (6.90) | 4 |
| Medication | 2 (13.33) | 2 | 9 (64.29) | 11 | 11 (37.93) | 13 |
| Non-pharmacological treatment | 0 (0.00) | 0 | 0 (0.00) | 0 | 0 (0.00) | 0 |
| Medication and non-pharmacological treatment | 0 (0.00) | 0 | 0 (0.00) | 0 | 0 (0.00) | 0 |
| **Outcome** |  |  |  |  |  |  |
| Recovered without sequelae | 2 (13.33) | 2 | 7 (50.00) | 12 | 9 (31.03) | 14 |
| Recovered with sequelae | 0 (0.00) | 0 | 0 (0.00) | 0 | 0 (0.00) | 0 |
| Adverse event ongoing | 0 (0.00) | 0 | 2 (14.29) | 2 | 2 (6.90) | 2 |
| Death | 0 (0.00) | 0 | 0 (0.00) | 0 | 0 (0.00) | 0 |
| Lost to follow-up | 1 (6.67) | 1 | 0 (0.00) | 0 | 1 (3.45) | 1 |

**a.** N (%): Number (percentage); calculated based on participants in each group; E: number of events
**b.** Participants with ≥2 types of adverse events were counted more than once

**Supplementary Table 4. Participant-level Listing of Device-related Adverse Events (Safety Population)**

| **Screening No.** | **Group** | **Adverse**  **Event** | **SOC** | **PT** | **Onset**  **Date** | **Resolution**  **Date** | **SAE** | **Severity** | **Causality** | **Action Taken for Investigational Device** | **Other Medical Treatment** | **Outcome** |
| --- | --- | --- | --- | --- | --- | --- | --- | --- | --- | --- | --- | --- |
| S09 | Intervention Group | Abnormal EEG | Investigations | Electroencephalogram abnormal | 2023-08-30 | NA | No | Mild | Possibly related | No action taken | None | Lost to follow-up |
| S10 | Control  Group | Contact dermatitis | Skin and subcutaneous tissue disorders | Dermatitis contact | 2023-08-20 | NA | No | Moderate | Probably related | Study discontinuation | Medication | Adverse event ongoing |
| S17 | Control  Group | Hyperactivity | Nervous system disorders | Psychomotor hyperactivity | 2023-10-16 | 2023-12-13 | No | Mild | Possibly related | Temporarily discontinued | None | Recovered without sequelae |
| S17 | Control  Group | Irritability | Psychiatric disorders | Irritability | 2023-10-16 | 2023-10-31 | No | Mild | Possibly related | Temporarily discontinued | None | Recovered without sequelae |
| S17 | Control  Group | Motor tic | Psychiatric disorders | Tic | 2023-10-16 | 2023-12-13 | No | Mild | Possibly related | Temporarily discontinued | None | Recovered without sequelae |
| S34 | Control  Group | Skin rash | Skin and subcutaneous tissue disorders | Rash | 2024-07-27 | 2024-08-19 | No | Mild | Probably related | Temporarily discontinued | Medication | Recovered without sequelae |

a. Adverse events were coded using System Organ Class (SOC) and Preferred Term (PT) classifications according to MedDRA version 27.

**Supplementary Table 5. Incidence of Device-related Adverse Events by System Organ Class (Safety Population)**

| **System Organ Class**  **Preferred Terms** | **Intervention**  **Group (N=15)** | | **Control**  **Group (N=14)** | | **Total (N=29)** | |
| --- | --- | --- | --- | --- | --- | --- |
|  | **N (%)** | **E** | **N (%)** | **E** | **N (%)** | **E** |
| Total | 1 (6.67) | 1 | 3 (21.43) | 5 | 4 (13.79) | 6 |
| p-value | 0.3295^&^ | | | |  |  |
| **Skin and subcutaneous tissue disorders** | **0 (0.00)** | **0** | **2 (14.29)** | **2** | **2 (6.90)** | **2** |
| Dermatitis contact | 0 (0.00) | 0 | 1 (7.14) | 1 | 1 (3.45) | 1 |
| Rash | 0 (0.00) | 0 | 1 (7.14) | 1 | 1 (3.45) | 1 |
| **Investigations** | **1 (6.67)** | **1** | **0 (0.00)** | **0** | **1 (3.45)** | **1** |
| Electroencephalogram abnormal | 1 (6.67) | 1 | 0 (0.00) | 0 | 1 (3.45) | 1 |
| **Nervous system disorders** | **0 (0.00)** | **0** | **1 (7.14)** | **1** | **1 (3.45)** | **1** |
| Psychomotor hyperactivity | 0 (0.00) | 0 | 1 (7.14) | 1 | 1 (3.45) | 1 |
| **Psychiatric disorders** | **0 (0.00)** | **0** | **1 (7.14)** | **2** | **1 (3.45)** | **2** |
| Irritability | 0 (0.00) | 0 | 1 (7.14) | 1 | 1 (3.45) | 1 |
| Tic | 0 (0.00) | 0 | 1 (7.14) | 1 | 1 (3.45) | 1 |

a. N (%): Number (percentage); calculated based on participants in each group; E: number of events.

b. Participants with ≥2 types of device-related adverse events were counted more than once.

c. Adverse events coded using MedDRA (v27.1) System Organ Class (SOC) and Preferred Term (PT).

^&^: Fisher’s exact test

**Supplementary Table 6. Changes in Vineland Adaptive Behavior Scale–II Scores From Baseline to Week 4 (ITT Population)**

| **Category** | **Intervention Group (N=14)** | **Control Group**  **(N=14)** | **Total**  **(N=28)** | **p-value** |
| --- | --- | --- | --- | --- |
| **Vineland Adaptive Behavior Scale-II** |  |  |  |  |
| **Domain Standard Score (points)** |  |  |  |  |
| **Communication** |  |  |  |  |
| **Baseline** |  |  |  | 0.8732^‡^ |
| N | 14 | 14 | 28 |  |
| Mean [SD] | 70.43 [8.39] | 70.93 [8.01] | 70.68 [8.06] |  |
| Median | 70.50 | 68.50 | 69.00 |  |
| Min, Max | 56.00, 86.00 | 59.00, 84.00 | 56.00, 86.00 |  |
| **Week 4** |  |  |  | 0.9403^‡^ |
| N | 13 | 12 | 25 |  |
| Mean [SD] | 71.15 [8.33] | 70.92 [7.23] | 71.04 [7.66] |  |
| Median | 71.00 | 72.50 | 71.00 |  |
| Min, Max | 54.00, 86.00 | 61.00, 79.00 | 54.00, 86.00 |  |
| **Change From Baseline (Week 4 – Baseline)** |  |  |  | 0.6198^#^ |
| N | 13 | 12 | 25 |  |
| Mean [SD] | 1.54 [4.10] | 0.83 [5.31] | 1.20 [4.63] |  |
| Median | 0.00 | 1.00 | 0.00 |  |
| Min, Max | -2.00, 10.00 | -6.00, 10.00 | -6.00, 10.00 |  |
| p-value | 0.2051^§^ | 0.5972^*^ | 0.2071^*^ |  |
| **Daily Living Skills** |  |  |  |  |
| **Baseline** |  |  |  | 0.2771^‡^ |
| N | 14 | 14 | 28 |  |
| Mean [SD] | 71.64 [6.82] | 75.14 [9.62] | 73.39 [8.38] |  |
| Median | 73.00 | 73.50 | 73.00 |  |
| Min, Max | 57.00, 82.00 | 59.00, 89.00 | 57.00, 89.00 |  |
| **Week 4** |  |  |  | 0.2238^‡^ |
| N | 13 | 12 | 25 |  |
| Mean [SD] | 72.23 [7.50] | 76.67 [10.15] | 74.36 [8.97] |  |
| Median | 72.00 | 78.50 | 74.00 |  |
| Min, Max | 53.00, 82.00 | 63.00, 94.00 | 53.00, 94.00 |  |
| **Change From Baseline (Week 4 – Baseline)** |  |  |  | 0.9401^‡^ |
| N | 13 | 12 | 25 |  |
| Mean [SD] | 0.92 [4.05] | 0.75 [7.06] | 0.84 [5.57] |  |
| Median | 0.00 | 1.00 | 0.00 |  |
| Min, Max | -4.00, 8.00 | -9.00, 13.00 | -9.00, 13.00 |  |
| p-value | 0.4273^*^ | 0.7198^*^ | 0.4584^*^ |  |
| **Socialization** |  |  |  |  |
| **Baseline** |  |  |  | 0.2767^‡^ |
| N | 14 | 14 | 28 |  |
| Mean [SD] | 62.93 [8.70] | 66.64 [8.99] | 64.79 [8.88] |  |
| Median | 64.50 | 69.00 | 66.00 |  |
| Min, Max | 49.00, 75.00 | 52.00, 81.00 | 49.00, 81.00 |  |
| **Week 4** |  |  |  | 0.7111^‡^ |
| N | 13 | 12 | 25 |  |
| Mean [SD] | 66.77 [9.04] | 68.25 [10.70] | 67.48 [9.69] |  |
| Median | 68.00 | 70.00 | 68.00 |  |
| Min, Max | 50.00, 79.00 | 52.00, 90.00 | 50.00, 90.00 |  |
| **Change From Baseline (Week 4 – Baseline)** |  |  |  | 0.1268^‡^ |
| N | 13 | 12 | 25 |  |
| Mean [SD] | 4.54 [5.65] | 0.92 [5.78] | 2.80 [5.89] |  |
| Median | 5.00 | 0.00 | 3.00 |  |
| Min, Max | -7.00, 16.00 | -7.00, 13.00 | -7.00, 16.00 |  |
| p-value | **0.0134^*^** | 0.5934^*^ | **0.0257^*^** |  |
| **Motor Skills** |  |  |  |  |
| **Baseline** |  |  |  | NA |
| N | NA | NA | NA |  |
| Mean [SD] | NA | NA | NA |  |
| Median | NA | NA | NA |  |
| Min, Max | NA | NA | NA |  |
| **Week 4** |  |  |  | NA |
| N | NA | NA | NA |  |
| Mean [SD] | NA | NA | NA |  |
| Median | NA | NA | NA |  |
| Min, Max | NA | NA | NA |  |
| **Change From Baseline (Week 4 – Baseline)** |  |  |  | NA |
| N | NA | NA | NA |  |
| Mean [SD] | NA | NA | NA |  |
| Median | NA | NA | NA |  |
| Min, Max | NA | NA | NA |  |
| p-value | NA | NA | NA |  |
| **Adaptive Behavior Composite Standard Score** |  |  |  |  |
| **Baseline** |  |  |  | 0.3717^‡^ |
| N | 14 | 14 | 28 |  |
| Mean [SD] | 205.00 [21.28] | 212.71 [23.57] | 208.86 [22.38] |  |
| Median | 207.50 | 217.00 | 210.00 |  |
| Min, Max | 162.00, 238.00 | 172.00, 250.00 | 162.00, 250.00 |  |
| **Week 4** |  |  |  | 0.5602^‡^ |
| N | 13 | 12 | 25 |  |
| Mean [SD] | 210.15 [22.59] | 215.83 [25.44] | 212.88 [23.67] |  |
| Median | 214.00 | 221.00 | 220.00 |  |
| Min, Max | 157.00, 239.00 | 176.00, 259.00 | 157.00, 259.00 |  |
| **Change From Baseline (Week 4 – Baseline)** |  |  |  | 0.3498^‡^ |
| N | 13 | 12 | 25 |  |
| Mean [SD] | 7.00 [8.97] | 2.50 [14.22] | 4.84 [11.76] |  |
| Median | 5.00 | 3.50 | 5.00 |  |
| Min, Max | -5.00, 25.00 | -20.00, 23.00 | -20.00, 25.00 |  |
| p-value | **0.0157^*^** | 0.5549^*^ | 0.0506^*^ |  |
| **Adaptive Behavior Composite Score (points)** |  |  |  |  |
| **Baseline** |  |  |  | 0.3556^‡^ |
| N | 14 | 14 | 28 |  |
| Mean [SD] | 64.50 [6.96] | 67.14 [7.88] | 65.82 [7.42] |  |
| Median | 65.50 | 68.50 | 66.00 |  |
| Min, Max | 51.00, 76.00 | 54.00, 80.00 | 51.00, 80.00 |  |
| **Week 4** |  |  |  | 0.5296^‡^ |
| N | 13 | 12 | 25 |  |
| Mean [SD] | 66.15 [7.40] | 68.17 [8.36] | 67.12 [7.78] |  |
| Median | 67.00 | 70.00 | 69.00 |  |
| Min, Max | 49.00, 76.00 | 55.00, 83.00 | 49.00, 83.00 |  |
| **Change From Baseline (Week 4 – Baseline)** |  |  |  | 0.3492^‡^ |
| N | 13 | 12 | 25 |  |
| Mean [SD] | 2.23 [2.86] | 0.75 [4.73] | 1.52 [3.86] |  |
| Median | 2.00 | 1.00 | 1.00 |  |
| Min, Max | -2.00, 8.00 | -6.00, 8.00 | -6.00, 8.00 |  |
| p-value | **0.0157^*^** | 0.5939^*^ | 0.0608^*^ |  |
| **Maladaptive Behavior Index (points)** |  |  |  |  |
| **Baseline** |  |  |  | 0.7667^‡^ |
| N | 14 | 14 | 28 |  |
| Mean [SD] | 20.07 [1.77] | 19.79 [3.09] | 19.93 [2.48] |  |
| Median | 20.00 | 20.00 | 20.00 |  |
| Min, Max | 17.00, 23.00 | 15.00, 24.00 | 15.00, 24.00 |  |
| **Week 4** |  |  |  | 0.3736^‡^ |
| N | 13 | 12 | 25 |  |
| Mean [SD] | 18.69 [2.36] | 19.75 [3.41] | 19.20 [2.90] |  |
| Median | 18.00 | 20.50 | 20.00 |  |
| Min, Max | 15.00, 23.00 | 13.00, 24.00 | 13.00, 24.00 |  |
| **Change From Baseline (Week 4 – Baseline)** |  |  |  | **0.0172^#^** |
| N | 13 | 12 | 25 |  |
| Mean [SD] | -1.38 [1.39] | 0.08 [1.44] | -0.68 [1.57] |  |
| Median | -1.00 | 0.00 | -1.00 |  |
| Min, Max | -4.00, 1.00 | -2.00, 3.00 | -4.00, 3.00 |  |
| p-value | **0.0044^§^** | 0.8451^*^ | **0.0409^*^** |  |

**a. Week 4 data for the Vineland Adaptive Behavior Scale-II were missing for participants S10, S20, and S28 and could not be imputed using the last observation carried forward (LOCF) method.**

**b. The Motor Skills domain, designed for children under 7 years of age, was not assessed or analyzed in this study, as all participants were 7 years or older in accordance with inclusion criterion 1 (children aged ≥7 and <12 years)**

**‡: Independent two-sample t-test**

**^#^: Wilcoxon rank sum test**

**^*^: Paired t-test**

**^§^: Wilcoxon signed rank test**

**-: Not analyzed**

Supplementary Table 7. Changes in Social Responsiveness Scale–2 (SRS-2) Scores From Baseline to Weeks 2 and 4 (ITT Population)

| **Category** | **Intervention Group (N=14)** | **Control Group**  **(N=14)** | **Total**  **(N=28)** | **p-value** |
| --- | --- | --- | --- | --- |
| **SRS-2 Total Score (Points)** |  |  |  |  |
| Baseline |  |  |  | 0.4775^‡^ |
| N | 14 | 14 | 28 |  |
| Mean (SD) | 149.21 (32.92) | 139.79 (36.23) | 144.50 (34.31) |  |
| Median | 154.50 | 143.50 | 147.50 |  |
| Min, Max | 75.00, 217.00 | 72.00, 204.00 | 72.00, 217.00 |  |
| **Week 2** |  |  |  | 0.8727^‡^ |
| N | 14 | 14 | 28 |  |
| Mean (SD) | 143.14 (36.73) | 140.86 (38.02) | 142.00 (36.70) |  |
| Median | 148.50 | 134.00 | 141.50 |  |
| Min, Max | 65.00, 213.00 | 79.00, 211.00 | 65.00, 213.00 |  |
| **Change From Baseline**  **(Week 2 – Baseline)** |  |  |  | 0.1580^‡^ |
| N | 14 | 14 | 28 |  |
| Mean (SD) | -6.07 (14.75) | 1.07 (10.98) | -2.50 (13.27) |  |
| Median | -6.50 | 5.00 | -1.00 |  |
| Min, Max | -29.00, 26.00 | -23.00, 17.00 | -29.00, 26.00 |  |
| p-value | 0.1474^*^ | 0.7209^*^ | 0.3275^*^ |  |
| **Week 4** |  |  |  | 0.9271^‡^ |
| N | 14 | 14 | 28 |  |
| Mean (SD) | 137.14 (31.40) | 138.36 (37.89) | 137.75 (34.15) |  |
| Median | 143.00 | 135.50 | 140.00 |  |
| Min, Max | 65.00, 197.00 | 77.00, 212.00 | 65.00, 212.00 |  |
| **Change From Baseline**  **(Week 4 – Baseline)** |  |  |  | **0.0250^‡^** |
| N | 14 | 14 | 28 |  |
| Mean (SD) | -12.07 (12.65) | -1.43 (10.96) | -6.75 (12.82) |  |
| Median | -12.00 | 1.00 | -9.00 |  |
| Min, Max | -32.00, 11.00 | -23.00, 12.00 | -32.00, 12.00 |  |
| p-value | **0.0034^*^** | 0.6339^*^ | **0.0096^*^** |  |

‡: Independent two-sample t-test

^*^: Paired t-test

**Supplementary Table 8. Changes in Children's Color Trails Test 1 and 2 (CCTT-1 and CCTT-2) Scores from Baseline to Weeks 2 and 4 (ITT Set)**

| **Category** | **Intervention Group (N=14)** | **Control Group**  **(N=14)** | **Total**  **(N=28)** | **p-value** |
| --- | --- | --- | --- | --- |
| **CCTT 1 Completion Time (seconds)** |  |  |  |  |
| **Baseline** |  |  |  | 0.6784^#^ |
| N | 14 | 14 | 28 |  |
| Mean (SD) | 24.57 (7.17) | 26.14 (16.15) | 25.36 (12.29) |  |
| Median | 24.00 | 19.50 | 21.50 |  |
| Min, Max | 15.00, 38.00 | 12.00, 76.00 | 12.00, 76.00 |  |
| **Week 2** |  |  |  | 0.1893^#^ |
| N | 14 | 14 | 28 |  |
| Mean (SD) | 26.29 (12.26) | 20.21 (7.14) | 23.25 (10.32) |  |
| Median | 22.50 | 19.00 | 20.50 |  |
| Min, Max | 13.00, 52.00 | 11.00, 38.00 | 11.00, 52.00 |  |
| **Change From Baseline**  **(Week 2 – Baseline)** |  |  |  | 0.1346^#^ |
| N | 14 | 14 | 28 |  |
| Mean (SD) | 1.71 (7.83) | -5.93 (16.19) | -2.11 (13.07) |  |
| Median | 0.00 | -3.00 | -1.00 |  |
| Min, Max | -10.00, 19.00 | -58.00, 8.00 | -58.00, 19.00 |  |
| p-value | 0.4274^*^ | 0.1989^§^ | 0.5192^§^ |  |
| **Week 4** |  |  |  | **0.0185^‡^** |
| N | 14 | 14 | 28 |  |
| Mean (SD) | 26.43 (10.26) | 18.64 (4.65) | 22.54 (8.76) |  |
| Median | 24.00 | 19.00 | 20.00 |  |
| Min, Max | 13.00, 48.00 | 12.00, 28.00 | 12.00, 48.00 |  |
| **Change From Baseline**  **(Week 4 – Baseline)** |  |  |  | **0.0361^#^** |
| N | 14 | 14 | 28 |  |
| Mean (SD) | 1.86 (7.72) | -7.50 (13.93) | -2.82 (12.03) |  |
| Median | 3.00 | -2.50 | 0.00 |  |
| Min, Max | -14.00, 15.00 | -48.00, 5.00 | -48.00, 15.00 |  |
| p-value | 0.3847^*^ | 0.0537^§^ | 0.4780^§^ |  |
| **CCTT 2 Completion Time (seconds)** |  |  |  |  |
| **Baseline** |  |  |  | 0.1538^#^ |
| N | 14 | 14 | 28 |  |
| Mean (SD) | 60.86 (33.59) | 44.36 (12.18) | 52.61 (26.18) |  |
| Median | 50.50 | 43.00 | 46.00 |  |
| Min, Max | 33.00, 161.00 | 26.00, 64.00 | 26.00, 161.00 |  |
| **Week 2** |  |  |  | 0.3221^#^ |
| N | 14 | 14 | 28 |  |
| Mean (SD) | 49.86 (16.92) | 45.36 (18.75) | 47.61 (17.68) |  |
| Median | 50.00 | 43.00 | 44.00 |  |
| Min, Max | 30.00, 94.00 | 23.00, 86.00 | 23.00, 94.00 |  |
| **Change From Baseline**  **(Week 2 – Baseline)** |  |  |  | 0.1973^#^ |
| N | 14 | 14 | 28 |  |
| Mean (SD) | -11.00 (18.52) | 1.00 (18.50) | -5.00 (19.16) |  |
| Median | -7.00 | -4.00 | -5.00 |  |
| Min, Max | -67.00, 10.00 | -18.00, 47.00 | -67.00, 47.00 |  |
| p-value | **0.0254^§^** | 0.8428^*^ | 0.1041^§^ |  |
| **Week 4** |  |  |  | 0.5044^#^ |
| N | 14 | 14 | 28 |  |
| Mean (SD) | 45.86 (17.52) | 41.21 (13.51) | 43.54 (15.53) |  |
| Median | 41.50 | 40.00 | 41.00 |  |
| Min, Max | 24.00, 91.00 | 23.00, 80.00 | 23.00, 91.00 |  |
| **Change From Baseline**  **(Week 4 – Baseline)** |  |  |  | 0.1288^#^ |
| N | 14 | 14 | 28 |  |
| Mean (SD) | -15.00 (19.78) | -3.14 (11.32) | -9.07 (16.93) |  |
| Median | -13.50 | -4.00 | -5.00 |  |
| Min, Max | -70.00, 11.00 | -28.00, 16.00 | -70.00, 16.00 |  |
| p-value | **0.0037^§^** | 0.3178^*^ | **0.0022^§^** |  |
| **Interference Index** |  |  |  |  |
| Baseline |  |  |  | 0.2788^#^ |
| N | 14 | 14 | 28 |  |
| Mean (SD) | 1.50 (1.10) | 1.16 (1.13) | 1.33 (1.11) |  |
| Median | 1.25 | 0.75 | 1.20 |  |
| Min, Max | 0.60, 4.80 | 0.00, 3.90 | 0.00, 4.80 |  |
| **Week 2** |  |  |  | 0.3450^#^ |
| N | 14 | 14 | 28 |  |
| Mean (SD) | 1.09 (0.76) | 1.40 (1.03) | 1.24 (0.90) |  |
| Median | 1.00 | 1.25 | 1.00 |  |
| Min, Max | 0.20, 2.80 | 0.20, 3.80 | 0.20, 3.80 |  |
| **Change From Baseline**  **(Week 2 – Baseline)** |  |  |  | 0.2060^#^ |
| N | 14 | 14 | 28 |  |
| Mean (SD) | -0.41 (1.14) | 0.24 (1.44) | -0.09 (1.31) |  |
| Median | -0.30 | 0.15 | -0.10 |  |
| Min, Max | -3.80, 1.40 | -2.00, 3.80 | -3.80, 3.80 |  |
| p-value | 0.1007^§^ | 0.5377^*^ | 0.5188^§^ |  |
| **Week 4** |  |  |  | 0.0602^‡^ |
| N | 14 | 14 | 28 |  |
| Mean (SD) | 0.81 (0.51) | 1.28 (0.72) | 1.05 (0.66) |  |
| Median | 0.70 | 1.10 | 0.95 |  |
| Min, Max | 0.20, 2.00 | 0.10, 2.60 | 0.10, 2.60 |  |
| **Change From Baseline**  **(Week 4 – Baseline)** |  |  |  | 0.0728^#^ |
| N | 14 | 14 | 28 |  |
| Mean (SD) | -0.69 (1.04) | 0.12 (1.12) | -0.28 (1.14) |  |
| Median | -0.55 | 0.00 | -0.35 |  |
| Min, Max | -3.50, 1.40 | -1.80, 2.00 | -3.50, 2.00 |  |
| p-value | **0.0079^§^** | 0.6914^*^ | 0.2001^*^ |  |
| **Difference Interference Score** |  |  |  |  |
| **Baseline** |  |  |  | 0.1291^#^ |
| N | 14 | 14 | 28 |  |
| Mean (SD) | 36.29 (30.98) | 18.21 (21.18) | 27.25 (27.62) |  |
| Median | 27.00 | 21.00 | 24.00 |  |
| Min, Max | 13.00, 133.00 | -37.00, 47.00 | -37.00, 133.00 |  |
| **Week 2** |  |  |  | 0.9449^#^ |
| N | 14 | 14 | 28 |  |
| Mean (SD) | 23.57 (12.05) | 25.14 (16.98) | 24.36 (14.47) |  |
| Median | 21.50 | 21.00 | 21.00 |  |
| Min, Max | 11.00, 47.00 | 3.00, 68.00 | 3.00, 68.00 |  |
| **Change From Baseline**  **(Week 2 – Baseline)** |  |  |  | 0.1352^#^ |
| N | 14 | 14 | 28 |  |
| Mean (SD) | -12.71 (23.56) | 6.93 (32.62) | -2.89 (29.66) |  |
| Median | -6.50 | 0.50 | -5.00 |  |
| Min, Max | -86.00, 12.00 | -25.00, 105.00 | -86.00, 105.00 |  |
| p-value | **0.0304^§^** | 0.8425^§^ | 0.1799^§^ |  |
| **Week 4** |  |  |  | 0.3696^#^ |
| N | 14 | 14 | 28 |  |
| Mean (SD) | 19.43 (12.78) | 22.57 (12.88) | 21.00 (12.69) |  |
| Median | 15.50 | 20.50 | 19.50 |  |
| Min, Max | 7.00, 51.00 | 4.00, 58.00 | 4.00, 58.00 |  |
| **Change From Baseline**  **(Week 4 – Baseline)** |  |  |  | **0.0156^#^** |
| N | 14 | 14 | 28 |  |
| Mean (SD) | -16.86 (22.25) | 4.36 (19.23) | -6.25 (23.09) |  |
| Median | -12.50 | 1.00 | -6.00 |  |
| Min, Max | -82.00, 15.00 | -28.00, 41.00 | -82.00, 41.00 |  |
| p-value | **0.0034^§^** | 0.4119^*^ | 0.1445^§^ |  |

**‡: Independent two-sample t-test**

**#: Wilcoxon rank sum test**

***: Paired t-test**

**§: Wilcoxon signed rank test**

**Supplementary Table 9. Changes in Stroop Color–Word Test Performance From Baseline to Week 2 and Week 4 (ITT Set)**

| **Category** | **Intervention Group (N=14)** | **Control Group**  **(N=14)** | **Total**  **(N=28)** | **p-value** |
| --- | --- | --- | --- | --- |
| **Word Reading Score** |  |  |  |  |
| **Baseline** |  |  |  | 0.6301^‡^ |
| N | 14 | 14 | 28 |  |
| Mean (SD) | 59.14 (8.49) | 57.07 (13.45) | 58.11 (11.09) |  |
| Median | 60.00 | 60.50 | 60.00 |  |
| Min, Max | 45.00, 74.00 | 24.00, 78.00 | 24.00, 78.00 |  |
| **Week 2** |  |  |  | 0.5968^‡^ |
| N | 14 | 14 | 28 |  |
| Mean (SD) | 63.00 (11.42) | 65.64 (14.51) | 64.32 (12.88) |  |
| Median | 62.50 | 67.50 | 64.00 |  |
| Min, Max | 39.00, 78.00 | 41.00, 87.00 | 39.00, 87.00 |  |
| **Change From Baseline**  **(Week 2 – Baseline)** |  |  |  | 0.0506^‡^ |
| N | 14 | 14 | 28 |  |
| Mean (SD) | 3.86 (6.42) | 8.57 (5.73) | 6.21 (6.44) |  |
| Median | 4.50 | 10.00 | 7.50 |  |
| Min, Max | -7.00, 13.00 | -3.00, 17.00 | -7.00, 17.00 |  |
| p-value | **0.0425^*^** | **<0.0001^*^** | **<0.0001^*^** |  |
| **Week 4** |  |  |  | 0.6944^‡^ |
| N | 14 | 14 | 28 |  |
| Mean (SD) | 64.86 (13.22) | 66.79 (12.46) | 65.82 (12.64) |  |
| Median | 63.00 | 65.50 | 63.00 |  |
| Min, Max | 46.00, 88.00 | 48.00, 87.00 | 46.00, 88.00 |  |
| **Change From Baseline**  **(Week 4 – Baseline)** |  |  |  | 0.3070^‡^ |
| N | 14 | 14 | 28 |  |
| Mean (SD) | 5.71 (9.79) | 9.71 (10.51) | 7.71 (10.17) |  |
| Median | 5.50 | 10.00 | 8.50 |  |
| Min, Max | -10.00, 18.00 | -12.00, 34.00 | -12.00, 34.00 |  |
| p-value | **0.0480^*^** | **0.0042^*^** | **0.0004^*^** |  |
| **Color Naming Score** |  |  |  |  |
| **Baseline** |  |  |  | 0.3711^‡^ |
| N | 14 | 14 | 28 |  |
| Mean (SD) | 42.50 (11.78) | 46.00 (8.26) | 44.25 (10.14) |  |
| Median | 42.00 | 43.50 | 43.00 |  |
| Min, Max | 20.00, 61.00 | 32.00, 61.00 | 20.00, 61.00 |  |
| **Week 2** |  |  |  | 0.5994^‡^ |
| N | 14 | 14 | 28 |  |
| Mean (SD) | 46.79 (12.34) | 48.93 (8.67) | 47.86 (10.52) |  |
| Median | 47.50 | 46.50 | 47.00 |  |
| Min, Max | 26.00, 68.00 | 35.00, 62.00 | 26.00, 68.00 |  |
| **Change From Baseline**  **(Week 2 – Baseline)** |  |  |  | 0.4248^‡^ |
| N | 14 | 14 | 28 |  |
| Mean (SD) | 4.29 (4.16) | 2.93 (4.68) | 3.61 (4.40) |  |
| Median | 5.00 | 3.00 | 4.00 |  |
| Min, Max | -4.00, 9.00 | -7.00, 11.00 | -7.00, 11.00 |  |
| p-value | **0.0020^*^** | **0.0358^*^** | **0.0002^*^** |  |
| **Week 4** |  |  |  | 0.7519^‡^ |
| N | 14 | 14 | 28 |  |
| Mean (SD) | 48.00 (13.99) | 49.43 (9.16) | 48.71 (11.63) |  |
| Median | 47.50 | 49.50 | 48.50 |  |
| Min, Max | 26.00, 76.00 | 37.00, 65.00 | 26.00, 76.00 |  |
| **Change From Baseline**  **(Week 4 – Baseline)** |  |  |  | 0.4437^‡^ |
| N | 14 | 14 | 28 |  |
| Mean (SD) | 5.50 (6.24) | 3.43 (7.77) | 4.46 (6.99) |  |
| Median | 5.50 | 3.00 | 4.00 |  |
| Min, Max | -9.00, 16.00 | -8.00, 17.00 | -9.00, 17.00 |  |
| p-value | **0.0057^*^** | 0.1228^*^ | **0.0022^*^** |  |
| **Color–Word Score** |  |  |  |  |
| **Baseline** |  |  |  | 0.4816^‡^ |
| **N** | 14 | 14 | 28 |  |
| **Mean (SD)** | 24.07 (7.32) | 26.07 (7.50) | 25.07 (7.34) |  |
| **Median** | 25.00 | 25.00 | 25.00 |  |
| **Min, Max** | 9.00, 33.00 | 12.00, 44.00 | 9.00, 44.00 |  |
| **Week 2** |  |  |  | 0.2325^‡^ |
| **N** | 14 | 14 | 28 |  |
| **Mean (SD)** | 27.36 (6.90) | 31.36 (10.11) | 29.36 (8.74) |  |
| **Median** | 28.50 | 28.00 | 28.50 |  |
| **Min, Max** | 17.00, 39.00 | 18.00, 55.00 | 17.00, 55.00 |  |
| **Change From Baseline**  **(Week 2 – Baseline)** |  |  |  | 0.3529^‡^ |
| **N** | 14 | 14 | 28 |  |
| **Mean (SD)** | 3.29 (6.21) | 5.29 (4.91) | 4.29 (5.58) |  |
| **Median** | 3.50 | 4.50 | 4.00 |  |
| **Min, Max** | -13.00, 13.00 | -2.00, 14.00 | -13.00, 14.00 |  |
| **p-value** | 0.0692^*^ | **0.0014^*^** | **<0.0001^§^** |  |
| **Week 4** |  |  |  | 0.1274^‡^ |
| N | 14 | 14 | 28 |  |
| Mean (SD) | 30.36 (9.48) | 35.93 (9.24) | 33.14 (9.61) |  |
| Median | 32.50 | 35.00 | 33.00 |  |
| Min, Max | 14.00, 44.00 | 23.00, 55.00 | 14.00, 55.00 |  |
| **Change From Baseline**  **(Week 4 – Baseline)** |  |  |  | 0.0778^‡^ |
| N | 14 | 14 | 28 |  |
| Mean (SD) | 6.29 (5.62) | 9.86 (4.62) | 8.07 (5.37) |  |
| Median | 5.50 | 10.00 | 8.50 |  |
| Min, Max | -2.00, 18.00 | 3.00, 18.00 | -2.00, 18.00 |  |
| p-value | **0.0011^*^** | **<0.0001^*^** | **<0.0001^*^** |  |
| **Inference Score** |  |  |  |  |
| **Baseline** |  |  |  | 0.5602^‡^ |
| N | 14 | 14 | 28 |  |
| Mean (SD) | 18.43 (8.64) | 19.93 (3.85) | 19.18 (6.61) |  |
| Median | 17.00 | 19.50 | 19.00 |  |
| Min, Max | 3.00, 35.00 | 14.00, 27.00 | 3.00, 35.00 |  |
| **Week 2** |  |  |  | 0.3887^‡^ |
| **N** | 14 | 14 | 28 |  |
| Mean (SD) | 19.43 (6.20) | 17.57 (4.94) | 18.50 (5.58) |  |
| Median | 19.50 | 17.50 | 19.00 |  |
| Min, Max | 8.00, 29.00 | 7.00, 25.00 | 7.00, 29.00 |  |
| **Change From Baseline**  **(Week 2 – Baseline)** |  |  |  | 0.1456^‡^ |
| N | 14 | 14 | 28 |  |
| Mean (SD) | 1.00 (7.13) | -2.36 (4.40) | -0.68 (6.06) |  |
| Median | 0.00 | -1.50 | -1.00 |  |
| Min, Max | -10.00, 17.00 | -10.00, 4.00 | -10.00, 17.00 |  |
| p-value | 0.6083^*^ | 0.0661^*^ | 0.5582^*^ |  |
| **Week 4** |  |  |  | 0.0935^‡^ |
| N | 14 | 14 | 28 |  |
| Mean (SD) | 17.64 (6.13) | 13.50 (6.45) | 15.57 (6.53) |  |
| Median | 15.50 | 14.00 | 15.00 |  |
| Min, Max | 10.00, 32.00 | 1.00, 23.00 | 1.00, 32.00 |  |
| **Change From Baseline**  **(Week 4 – Baseline)** |  |  |  | **0.0433^‡^** |
| N | 14 | 14 | 28 |  |
| Mean (SD) | -0.79 (7.80) | -6.43 (6.16) | -3.61 (7.47) |  |
| Median | 0.00 | -6.00 | -3.50 |  |
| Min, Max | -16.00, 10.00 | -19.00, 3.00 | -19.00, 10.00 |  |
| p-value | 0.7122^*^ | **0.0018^*^** | **0.0166^*^** |  |

**‡: Independent two-sample t-test**

**^*^: Paired t-test**

**^§^: Wilcoxon signed rank test**

**Supplementary Table 10. Changes in the Korean version of the Children’s Sleep Habits Questionnaire (K-CSHQ) from Baseline to Weeks 2 and 4 (ITT population)**

| **Category** | **Intervention Group (N=14)** | **Control**  **Group**  **(N=14)** | **Total**  **(N=28)** | **p-value** |
| --- | --- | --- | --- | --- |
| **K-CSHQ Total Score (points)** |  |  |  |  |
| **Baseline** |  |  |  | 0.8741^‡^ |
| N | 14 | 14 | 28 |  |
| Mean (SD) | 65.07 (8.33) | 64.57 (8.20) | 64.82 (8.12) |  |
| Median | 63.50 | 62.50 | 62.50 |  |
| Min, Max | 56.00, 81.00 | 52.00, 83.00 | 52.00, 83.00 |  |
| **Week 2** |  |  |  | 0.5698^‡^ |
| N | 14 | 14 | 28 |  |
| Mean (SD) | 64.14 (6.67) | 65.79 (8.34) | 64.96 (7.46) |  |
| Median | 65.00 | 64.00 | 64.00 |  |
| Min, Max | 56.00, 77.00 | 52.00, 84.00 | 52.00, 84.00 |  |
| **Change From Baseline**  **(Week 2 – Baseline)** |  |  |  | **0.0475^#^** |
| N | 14 | 14 | 28 |  |
| Mean (SD) | -0.93 (4.29) | 1.21 (1.93) | 0.14 (3.44) |  |
| Median | -1.00 | 1.00 | 0.00 |  |
| Min, Max | -13.00, 5.00 | -2.00, 5.00 | -13.00, 5.00 |  |
| p-value | 0.4604^§^ | **0.0349^*^** | 0.5741^§^ |  |
| **Week 4** |  |  |  | 0.9424^‡^ |
| N | 14 | 14 | 28 |  |
| Mean (SD) | 64.57 (6.36) | 64.36 (8.97) | 64.46 (7.63) |  |
| Median | 65.50 | 63.50 | 64.50 |  |
| Min, Max | 55.00, 72.00 | 52.00, 86.00 | 52.00, 86.00 |  |
| **Change From Baseline**  **(Week 4 – Baseline)** |  |  |  | 0.8848^‡^ |
| N | 14 | 14 | 28 |  |
| Mean (SD) | -0.50 (6.14) | -0.21 (3.96) | -0.36 (5.07) |  |
| Median | -1.50 | 0.00 | 0.00 |  |
| Min, Max | -11.00, 14.00 | -9.00, 6.00 | -11.00, 14.00 |  |
| p-value | 0.7653^*^ | 0.8429^*^ | 0.7123^*^ |  |

**‡: Independent two-sample t-test**

**^#^: Wilcoxon rank sum test**

**^*^: Paired t-test**

**^§^: Wilcoxon signed rank test**

**Supplementary Table 11. Changes in Aberrant Behavior Checklist-II (ABC-II) Scores From Baseline to Week 2 and Week 4 (ITT Set)**

| **Category** | **Intervention Group (N=14)** | **Control Group**  **(N=14)** | **Total**  **(N=28)** | **p-value** |
| --- | --- | --- | --- | --- |
| **ABC-II Total Score (points)** |  |  |  |  |
| **Baseline** |  |  |  | 0.5050^#^ |
| N | 14 | 14 | 28 |  |
| Mean (SD) | 18.79 (20.85) | 29.29 (28.23) | 24.04 (24.93) |  |
| Median | 12.50 | 25.00 | 13.50 |  |
| Min, Max | 5.00, 87.00 | 0.00, 95.00 | 0.00, 95.00 |  |
| **Week 2** |  |  |  | 0.6621^#^ |
| N | 14 | 14 | 28 |  |
| Mean (SD) | 24.21 (25.15) | 31.36 (28.15) | 27.79 (26.44) |  |
| Median | 18.50 | 20.50 | 19.00 |  |
| Min, Max | 2.00, 103.00 | 0.00, 84.00 | 0.00, 103.00 |  |
| **Change From Baseline**  **(Week 2 – Baseline)** |  |  |  | 0.3278^‡^ |
| N | 14 | 14 | 28 |  |
| Mean (SD) | 5.43 (9.25) | 2.07 (8.55) | 3.75 (8.91) |  |
| Median | 4.00 | 2.50 | 3.50 |  |
| Min, Max | -8.00, 24.00 | -14.00, 17.00 | -14.00, 24.00 |  |
| p-value | **0.0468^*^** | 0.3813^*^ | **0.0344^*^** |  |
| **Week 4** |  |  |  | 0.5198^#^ |
| N | 14 | 14 | 28 |  |
| Mean (SD) | 20.64 (21.84) | 31.71 (31.56) | 26.18 (27.22) |  |
| Median | 15.00 | 22.00 | 16.50 |  |
| Min, Max | 1.00, 90.00 | 0.00, 95.00 | 0.00, 95.00 |  |
| **Change From Baseline**  **(Week 4 – Baseline)** |  |  |  | 0.8741^‡^ |
| N | 14 | 14 | 28 |  |
| Mean (SD) | 1.86 (6.65) | 2.43 (11.59) | 2.14 (9.28) |  |
| Median | 3.50 | 0.00 | 1.00 |  |
| Min, Max | -11.00, 13.00 | -18.00, 26.00 | -18.00, 26.00 |  |
| p-value | 0.3154^*^ | 0.4470^*^ | 0.2321^*^ |  |

‡: Independent two-sample t-test

^#^: Wilcoxon rank sum test

^*^: Paired t-test

**Supplementary Table 12. Changes in Social Communication Questionnaire (SCQ) Scores From Baseline to Week 2 and Week 4 (ITT Set)**

| **Category** | **Intervention Group (N=14)** | **Control Group**  **(N=14)** | **Total**  **(N=28)** | **p-value** |
| --- | --- | --- | --- | --- |
| **SCQ Current Score (points)** |  |  |  |  |
| **Baseline** |  |  |  | 0.2877^‡^ |
| N | 14 | 14 | 28 |  |
| Mean (SD) | 11.79 (8.21) | 8.71 (6.68) | 10.25 (7.51) |  |
| Median | 9.50 | 7.50 | 8.50 |  |
| Min, Max | 2.00, 30.00 | 0.00, 22.00 | 0.00, 30.00 |  |
| **Week 2** |  |  |  | 0.7817^#^ |
| N | 14 | 14 | 28 |  |
| Mean (SD) | 10.57 (8.62) | 9.29 (7.82) | 9.93 (8.10) |  |
| Median | 7.00 | 7.50 | 7.00 |  |
| Min, Max | 2.00, 30.00 | 1.00, 26.00 | 1.00, 30.00 |  |
| **Change From Baseline**  **(Week 2 – Baseline)** |  |  |  | 0.2322^‡^ |
| N | 14 | 14 | 28 |  |
| Mean (SD) | -1.21 (4.51) | 0.57 (3.08) | -0.32 (3.90) |  |
| Median | -1.50 | 1.00 | -0.50 |  |
| Min, Max | -8.00, 11.00 | -5.00, 6.00 | -8.00, 11.00 |  |
| p-value | 0.3321^*^ | 0.5000^*^ | 0.6660^*^ |  |
| **Week 4** |  |  |  | 0.8060^‡^ |
| N | 14 | 14 | 28 |  |
| Mean (SD) | 8.36 (4.96) | 7.79 (7.05) | 8.07 (5.99) |  |
| Median | 7.00 | 4.50 | 6.50 |  |
| Min, Max | 1.00, 17.00 | 0.00, 21.00 | 0.00, 21.00 |  |
| **Change From Baseline**  **(Week 4 – Baseline)** |  |  |  | 0.1507^#^ |
| N | 14 | 14 | 28 |  |
| Mean (SD) | -3.43 (4.09) | -0.93 (3.08) | -2.18 (3.77) |  |
| Median | -2.00 | -1.00 | -2.00 |  |
| Min, Max | -13.00, 2.00 | -7.00, 6.00 | -13.00, 6.00 |  |
| p-value | **0.0032^§^** | 0.2790^*^ | **0.0012^§^** |  |
| **SCQ Lifetime Score (points)** |  |  |  |  |
| **Baseline** |  |  |  | 0.2456^‡^ |
| N | 14 | 14 | 28 |  |
| Mean (SD) | 21.29 (8.29) | 17.00 (10.66) | 19.14 (9.62) |  |
| Median | 22.00 | 13.00 | 18.00 |  |
| Min, Max | 8.00, 35.00 | 3.00, 34.00 | 3.00, 35.00 |  |
| **Week 2** |  |  |  | 0.1567^‡^ |
| N | 14 | 14 | 28 |  |
| Mean (SD) | 21.50 (8.05) | 16.36 (10.46) | 18.93 (9.52) |  |
| Median | 21.50 | 13.00 | 19.50 |  |
| Min, Max | 10.00, 35.00 | 4.00, 34.00 | 4.00, 35.00 |  |
| **Change From Baseline**  **(Week 2 – Baseline)** |  |  |  | 0.5455^‡^ |
| N | 14 | 14 | 28 |  |
| Mean (SD) | 0.21 (4.82) | -0.64 (1.95) | -0.21 (3.63) |  |
| Median | 0.00 | -0.50 | 0.00 |  |
| Min, Max | -6.00, 12.00 | -4.00, 3.00 | -6.00, 12.00 |  |
| p-value | 0.8705^*^ | 0.2382^*^ | 0.4848^§^ |  |
| **Week 4** |  |  |  | 0.3293^‡^ |
| N | 14 | 14 | 28 |  |
| Mean (SD) | 19.93 (7.34) | 16.50 (10.61) | 18.21 (9.12) |  |
| Median | 20.00 | 13.00 | 18.00 |  |
| Min, Max | 8.00, 29.00 | 5.00, 34.00 | 5.00, 34.00 |  |
| **Change From Baseline**  **(Week 4 – Baseline)** |  |  |  | 0.4962^‡^ |
| N | 14 | 14 | 28 |  |
| Mean (SD) | -1.36 (4.01) | -0.50 (2.35) | -0.93 (3.25) |  |
| Median | -1.50 | -0.50 | -1.00 |  |
| Min, Max | -8.00, 4.00 | -4.00, 4.00 | -8.00, 4.00 |  |
| p-value | 0.2278^*^ | 0.4394^*^ | 0.1426^*^ |  |

‡: Independent two-sample t-test

^#^: Wilcoxon rank sum test

^*^: Paired t-test

^§^: Wilcoxon signed rank test

**Supplementary Table 13. Changes in Korean ADHD Rating Scale (K-ARS) Scores From Baseline to Week 2 and Week 4 (ITT Set)**

| **Category** | **Intervention Group (N=14)** | **Control Group**  **(N=14)** | **Total**  **(N=28)** | **p-value** |
| --- | --- | --- | --- | --- |
| **K-ARS Total Score (points)** |  |  |  |  |
| **Baseline** |  |  |  | 0.8398^‡^ |
| N | 14 | 14 | 28 |  |
| Mean (SD) | 23.00 (12.59) | 22.00 (13.31) | 22.50 (12.73) |  |
| Median | 20.50 | 19.50 | 20.50 |  |
| Min, Max | 6.00, 52.00 | 2.00, 40.00 | 2.00, 52.00 |  |
| Week 2 |  |  |  | 0.8540^#^ |
| N | 14 | 14 | 28 |  |
| Mean (SD) | 19.36 (11.85) | 19.29 (13.11) | 19.32 (12.26) |  |
| Median | 17.00 | 18.00 | 17.00 |  |
| Min, Max | 6.00, 52.00 | 2.00, 39.00 | 2.00, 52.00 |  |
| Change From Baseline  (Week 2 – Baseline) |  |  |  | 0.3211^#^ |
| N | 14 | 14 | 28 |  |
| Mean (SD) | -3.64 (3.91) | -2.71 (6.02) | -3.18 (5.00) |  |
| Median | -3.50 | -1.00 | -2.00 |  |
| Min, Max | -9.00, 3.00 | -15.00, 4.00 | -15.00, 4.00 |  |
| p-value | **0.0041^*^** | 0.2234^§^ | **0.0023^*^** |  |
| Week 4 |  |  |  | 0.9770^‡^ |
| N | 14 | 14 | 28 |  |
| Mean (SD) | 20.50 (12.54) | 20.36 (13.36) | 20.43 (12.72) |  |
| Median | 17.50 | 18.50 | 18.50 |  |
| Min, Max | 7.00, 52.00 | 1.00, 39.00 | 1.00, 52.00 |  |
| Change From Baseline  (Week 4 – Baseline) |  |  |  | 0.5179^#^ |
| N | 14 | 14 | 28 |  |
| Mean (SD) | -2.50 (6.85) | -1.64 (5.88) | -2.07 (6.28) |  |
| Median | -2.00 | 0.00 | -0.50 |  |
| Min, Max | -19.00, 10.00 | -18.00, 4.00 | -19.00, 10.00 |  |
| p-value | 0.1951^*^ | 0.5889^§^ | 0.1557^§^ |  |

‡: Independent two-sample t-test

^#^: Wilcoxon rank sum test

^*^: Paired t-test

^§^: Wilcoxon signed rank test

**Supplementary Table 14. Changes in Korean Child Behavior Checklist (K-CBCL) Scores From Baseline to Week 2 and Week 4 (ITT Set)**

| **Category** | **Intervention Group (N=14)** | **Control Group**  **(N=14)** | **Total**  **(N=28)** | **p-value** |
| --- | --- | --- | --- | --- |
| **Social Competence Scale Score (points)** |  |  |  |  |
| Baseline |  |  |  | 0.5322^#^ |
| N | 14 | 14 | 28 |  |
| Mean (SD) | 34.50 (5.50) | 35.50 (10.14) | 35.00 (8.02) |  |
| Median | 33.50 | 32.00 | 33.00 |  |
| Min, Max | 27.00, 45.00 | 27.00, 66.00 | 27.00, 66.00 |  |
| Week 2 |  |  |  | 0.9265^#^ |
| N | 14 | 14 | 28 |  |
| Mean (SD) | 35.21 (7.88) | 36.29 (11.09) | 35.75 (9.45) |  |
| Median | 32.50 | 33.50 | 33.00 |  |
| Min, Max | 27.00, 52.00 | 23.00, 66.00 | 23.00, 66.00 |  |
| Change From Baseline  (Week 2 – Baseline) |  |  |  | 0.9690^‡^ |
| N | 14 | 14 | 28 |  |
| Mean (SD) | 0.71 (3.69) | 0.79 (5.73) | 0.75 (4.73) |  |
| Median | 0.00 | 0.00 | 0.00 |  |
| Min, Max | -6.00, 7.00 | -8.00, 14.00 | -8.00, 14.00 |  |
| p-value | 0.4815^*^ | 0.6163^*^ | 0.4085^*^ |  |
| Week 4 |  |  |  | 0.4879^#^ |
| N | 14 | 14 | 28 |  |
| Mean (SD) | 35.64 (8.17) | 35.50 (11.12) | 35.57 (9.57) |  |
| Median | 33.00 | 31.00 | 32.50 |  |
| Min, Max | 24.00, 52.00 | 23.00, 66.00 | 23.00, 66.00 |  |
| Change From Baseline  (Week 4 – Baseline) |  |  |  | 0.3867^#^ |
| N | 14 | 14 | 28 |  |
| Mean (SD) | 1.14 (3.84) | 0.00 (4.95) | 0.57 (4.38) |  |
| Median | 0.00 | 0.00 | 0.00 |  |
| Min, Max | -6.00, 7.00 | -8.00, 13.00 | -8.00, 13.00 |  |
| p-value | 0.2856^*^ | 0.7070^§^ | 0.4962^*^ |  |
| **Total Problem Behavior Score (points)** |  |  |  |  |
| Baseline |  |  |  | 0.8243^‡^ |
| N | 14 | 14 | 28 |  |
| Mean (SD) | 60.64 (11.22) | 61.64 (12.35) | 61.14 (11.59) |  |
| Median | 60.50 | 63.00 | 62.50 |  |
| Min, Max | 46.00, 89.00 | 30.00, 81.00 | 30.00, 89.00 |  |
| Week 2 |  |  |  | 0.1743^#^ |
| N | 14 | 14 | 28 |  |
| Mean (SD) | 57.93 (10.34) | 62.93 (12.72) | 60.43 (11.66) |  |
| Median | 58.50 | 62.50 | 59.00 |  |
| Min, Max | 44.00, 87.00 | 35.00, 81.00 | 35.00, 87.00 |  |
| Change From Baseline  (Week 2 – Baseline) |  |  |  | **0.0209^‡^** |
| N | 14 | 14 | 28 |  |
| Mean (SD) | -2.71 (4.71) | 1.29 (3.85) | -0.71 (4.69) |  |
| Median | -2.50 | 0.00 | -1.00 |  |
| Min, Max | -12.00, 7.00 | -4.00, 9.00 | -12.00, 9.00 |  |
| p-value | **0.0505^*^** | 0.2337^*^ | 0.4273^*^ |  |
| Week 4 |  |  |  | 0.3788^‡^ |
| N | 14 | 14 | 28 |  |
| Mean (SD) | 56.86 (12.02) | 61.29 (14.07) | 59.07 (13.04) |  |
| Median | 56.00 | 62.00 | 59.50 |  |
| Min, Max | 39.00, 89.00 | 30.00, 87.00 | 30.00, 89.00 |  |
| Change From Baseline  (Week 4 – Baseline) |  |  |  | 0.0890^‡^ |
| N | 14 | 14 | 28 |  |
| Mean (SD) | -3.79 (4.04) | -0.36 (6.03) | -2.07 (5.33) |  |
| Median | -3.00 | -0.50 | -2.00 |  |
| Min, Max | -10.00, 3.00 | -13.00, 11.00 | -13.00, 11.00 |  |
| p-value | **0.0039^*^** | 0.8282^*^ | **0.0496^*^** |  |

‡: Independent two-sample t-test

^#^: Wilcoxon rank sum test

^*^: Paired t-test

^§^: Wilcoxon signed rank test

**Supplementary Table 15. Changes in Short Sensory Profile-2 (SSP-2) Scores From Baseline to Week 2 and Week 4 (ITT Set)**

| **Category** | **Intervention Group (N=14)** | **Control Group**  **(N=14)** | **Total**  **(N=28)** | **p-value** |
| --- | --- | --- | --- | --- |
| **Sensory Seeking (Score)** |  |  |  |  |
| **Baseline** |  |  |  | 0.7128^‡^ |
| **N** | 14 | 14 | 28 |  |
| **Mean (SD)** | 15.71 (6.45) | 16.71 (7.71) | 16.21 (6.99) |  |
| **Median** | 14.00 | 15.50 | 14.00 |  |
| **Min, Max** | 7.00, 32.00 | 7.00, 33.00 | 7.00, 33.00 |  |
| **Week 2** |  |  |  | 0.8897^#^ |
| **N** | 14 | 14 | 28 |  |
| **Mean (SD)** | 14.21 (5.65) | 15.57 (6.81) | 14.89 (6.18) |  |
| **Median** | 12.50 | 13.50 | 12.50 |  |
| **Min, Max** | 7.00, 29.00 | 7.00, 28.00 | 7.00, 29.00 |  |
| **Change From Baseline**  **(Week 2 – Baseline)** |  |  |  | 0.6373^#^ |
| **N** | 14 | 14 | 28 |  |
| **Mean (SD)** | -1.50 (2.44) | -1.14 (1.88) | -1.32 (2.14) |  |
| **Median** | -1.50 | -0.50 | -1.00 |  |
| **Min, Max** | -6.00, 3.00 | -6.00, 1.00 | -6.00, 3.00 |  |
| **p-value** | **0.0388^*^** | **0.0273^§^** | **0.0030^*^** |  |
| **Week 4** |  |  |  | 0.3798^#^ |
| **N** | 14 | 14 | 28 |  |
| **Mean (SD)** | 13.86 (6.92) | 15.57 (7.42) | 14.71 (7.09) |  |
| **Median** | 13.00 | 16.50 | 13.00 |  |
| **Min, Max** | 7.00, 35.00 | 7.00, 28.00 | 7.00, 35.00 |  |
| **Change From Baseline**  **(Week 4 – Baseline)** |  |  |  | 0.5488^‡^ |
| **N** | 14 | 14 | 28 |  |
| **Mean (SD)** | -1.86 (3.88) | -1.14 (2.03) | -1.50 (3.06) |  |
| **Median** | -1.00 | -1.00 | -1.00 |  |
| **Min, Max** | -11.00, 3.00 | -5.00, 2.00 | -11.00, 3.00 |  |
| **p-value** | 0.0966^*^ | 0.0554^*^ | **0.0133^§^** |  |
| \| **Sensory Avoiding (Score)** \| \| --- \|  \|  \| \| --- \| |  |  |  |  |
| **Baseline** |  |  |  | 0.5969^‡^ |
| **N** | 14 | 14 | 28 |  |
| **Mean (SD)** | 22.07 (7.28) | 23.57 (7.54) | 22.82 (7.31) |  |
| **Median** | 20.00 | 25.00 | 23.50 |  |
| **Min, Max** | 12.00, 41.00 | 11.00, 38.00 | 11.00, 41.00 |  |
| **Week 2** |  |  |  | 0.6157^‡^ |
| **N** | 14 | 14 | 28 |  |
| **Mean (SD)** | 20.43 (7.66) | 21.86 (7.21) | 21.14 (7.34) |  |
| **Median** | 18.00 | 23.50 | 21.00 |  |
| **Min, Max** | 10.00, 40.00 | 10.00, 38.00 | 10.00, 40.00 |  |
| **Change From Baseline**  **(Week 2 – Baseline)** |  |  |  | 0.9492^‡^ |
| **N** | 14 | 14 | 28 |  |
| **Mean (SD)** | -1.64 (3.20) | -1.71 (2.64) | -1.68 (2.88) |  |
| **Median** | -2.00 | -2.00 | -2.00 |  |
| **Min, Max** | -7.00, 4.00 | -6.00, 3.00 | -7.00, 4.00 |  |
| **p-value** | 0.0770^*^ | **0.0305^*^** | **0.0047^*^** |  |
| **Week 4** |  |  |  | 0.3586^‡^ |
| **N** | 14 | 14 | 28 |  |
| **Mean (SD)** | 19.50 (8.06) | 22.36 (8.11) | 20.93 (8.07) |  |
| **Median** | 18.00 | 23.50 | 20.50 |  |
| **Min, Max** | 9.00, 41.00 | 9.00, 42.00 | 9.00, 42.00 |  |
| **Change From Baseline**  **(Week 4 – Baseline)** |  |  |  | 0.3211^#^ |
| **N** | 14 | 14 | 28 |  |
| **Mean (SD)** | -2.57 (4.60) | -1.21 (4.41) | -1.89 (4.47) |  |
| **Median** | -3.50 | -1.00 | -1.50 |  |
| **Min, Max** | -12.00, 5.00 | -13.00, 4.00 | -13.00, 5.00 |  |
| **p-value** | 0.0568^*^ | 0.4084^§^ | **0.0336^*^** |  |
| \| **Sensory Sensitivity (Score)** \| \| --- \|  \|  \| \| --- \| |  |  |  |  |
| **Baseline** |  |  |  | 0.7128^‡^ |
| **N** | 14 | 14 | 28 |  |
| **Mean (SD)** | 25.29 (7.99) | 26.43 (8.25) | 25.86 (7.99) |  |
| **Median** | 24.00 | 26.50 | 25.00 |  |
| **Min, Max** | 14.00, 45.00 | 14.00, 41.00 | 14.00, 45.00 |  |
| **Week 2** |  |  |  | 0.8559^‡^ |
| **N** | 14 | 14 | 28 |  |
| **Mean (SD)** | 24.86 (8.47) | 24.29 (8.00) | 24.57 (8.09) |  |
| **Median** | 23.00 | 24.00 | 24.00 |  |
| **Min, Max** | 13.00, 44.00 | 11.00, 39.00 | 11.00, 44.00 |  |
| **Change From Baseline**  **(Week 2 – Baseline)** |  |  |  | 0.1735^‡^ |
| **N** | 14 | 14 | 28 |  |
| **Mean (SD)** | -0.43 (3.39) | -2.14 (3.08) | -1.29 (3.30) |  |
| **Median** | -1.00 | -2.00 | -1.50 |  |
| **Min, Max** | -4.00, 8.00 | -7.00, 3.00 | -7.00, 8.00 |  |
| **p-value** | 0.6441^*^ | **0.0220^*^** | **0.0489^*^** |  |
| **Week 4** |  |  |  | 0.4613^#^ |
| **N** | 14 | 14 | 28 |  |
| **Mean (SD)** | 23.79 (8.76) | 24.79 (8.75) | 24.29 (8.61) |  |
| **Median** | 22.00 | 26.00 | 23.00 |  |
| **Min, Max** | 13.00, 49.00 | 12.00, 41.00 | 12.00, 49.00 |  |
| **Change From Baseline**  **(Week 4 – Baseline)** |  |  |  | 0.9362^‡^ |
| **N** | 14 | 14 | 28 |  |
| **Mean (SD)** | -1.50 (4.90) | -1.64 (4.45) | -1.57 (4.59) |  |
| **Median** | -2.00 | -1.50 | -2.00 |  |
| **Min, Max** | -12.00, 9.00 | -10.00, 8.00 | -12.00, 9.00 |  |
| **p-value** | 0.2722^*^ | 0.1903^*^ | 0.0812^*^ |  |
| \| **Sensory Registration (Score)** \| \| --- \|  \|  \| \| --- \| |  |  |  |  |
| **Baseline** |  |  |  | 0.9785^‡^ |
| **N** | 14 | 14 | 28 |  |
| **Mean (SD)** | 17.57 (6.97) | 17.64 (6.93) | 17.61 (6.82) |  |
| **Median** | 16.50 | 18.00 | 17.50 |  |
| **Min, Max** | 9.00, 36.00 | 8.00, 31.00 | 8.00, 36.00 |  |
| **Week 2** |  |  |  | 0.5334^#^ |
| **N** | 14 | 14 | 28 |  |
| **Mean (SD)** | 17.00 (6.09) | 18.36 (6.82) | 17.68 (6.38) |  |
| **Median** | 16.50 | 19.00 | 17.50 |  |
| **Min, Max** | 9.00, 34.00 | 8.00, 32.00 | 8.00, 34.00 |  |
| **Change From Baseline**  **(Week 2 – Baseline)** |  |  |  | 0.0944^#^ |
| **N** | 14 | 14 | 28 |  |
| **Mean (SD)** | -0.57 (2.24) | 0.71 (2.40) | 0.07 (2.37) |  |
| **Median** | -1.50 | 1.00 | 0.00 |  |
| **Min, Max** | -3.00, 4.00 | -5.00, 4.00 | -5.00, 4.00 |  |
| **p-value** | 0.4082^§^ | 0.2856^*^ | 0.8746^*^ |  |
| **Week 4** |  |  |  | 1.0000^#^ |
| **N** | 14 | 14 | 28 |  |
| **Mean (SD)** | 17.21 (7.28) | 16.79 (6.60) | 17.00 (6.82) |  |
| **Median** | 15.00 | 18.00 | 15.50 |  |
| **Min, Max** | 9.00, 38.00 | 8.00, 26.00 | 8.00, 38.00 |  |
| **Change From Baseline**  **(Week 4 – Baseline)** |  |  |  | 0.2550^#^ |
| **N** | 14 | 14 | 28 |  |
| **Mean (SD)** | -0.36 (4.73) | -0.86 (2.74) | -0.61 (3.80) |  |
| **Median** | 1.50 | -1.00 | 0.00 |  |
| **Min, Max** | -15.00, 4.00 | -6.00, 3.00 | -15.00, 4.00 |  |
| **p-value** | 0.7705^§^ | 0.2631^*^ | 0.8641^§^ |  |

**‡: Independent two-sample t-test**

**^#^: Wilcoxon rank sum test**

**^*^: Paired t-test**

**^§^: Wilcoxon signed rank test**

**Supplementary Table 16. Relative band power difference (Week 4 – Baseline) of frontal EEG**

|  | **Delta** | | **Theta** | | **Alpha** | | **Beta** | | **High-beta** | | **Gamma** | | **High-gamma** | | |
| --- | --- | --- | --- | --- | --- | --- | --- | --- | --- | --- | --- | --- | --- | --- | --- |
|  | **Intervention  Group (N=10)** | **Control Group (N=12)** | **Intervention  Group (N=10)** | **Control Group (N=12)** | **Intervention  Group (N=10)** | **Control Group (N=12)** | **Intervention  Group (N=10)** | **Control Group (N=12)** | **Intervention  Group (N=10)** | **Control Group (N=12)** | **Intervention  Group (N=10)** | **Control Group (N=12)** | **Intervention  Group (N=10)** | **Control Group (N=12)** | |
| **AF3** |  |  |  |  |  |  |  |  |  |  |  |  |  |  | |
| Mean (SD) | -6.7326 (13.3687) | 2.6101 (19.6867) | 5.3046 (6.8212) | -2.7768 (9.1049) | 4.9792 (6.999) | -1.8908 (11.4253) | -0.36281 (4.5849) | -0.036256 (2.9776) | -0.54181 (1.1999) | 0.52282 (1.776) | -1.3075 (2.555) | 0.92268 (3.0512) | -1.307 (2.4824) | 0.63049 (1.949) | |
| Median | -6.7448 | 4.3112 | 4.3946 | 0.32481 | 4.4135 | -2.4744 | 0.48236 | 0.30517 | -0.11823 | 0.30873 | -0.12244 | 0.31701 | -0.20123 | 0.1293 | |
| Min, Max | -28.3247, 20.3088 | -21.4608, 49.1798 | -0.43996, 20.1533 | -25.8498, 6.3109 | -3.0489, 22.0192 | -15.7395, 24.1892 | -12.7516, 3.8846 | -6.3527, 3.7298 | -3.7286, 0.41599 | -1.6844, 5.6519 | -8.0782, 0.33556 | -1.6693, 9.7397 | -7.8764, 0.089389 | -1.6223, 5.7517 | |
| p-value | 0.16016 | 1 | 0.064453 | 0.79102 | **0.027344** | 0.46973 | 0.27539 | 0.90967 | 0.19336 | 0.33936 | 0.10547 | 0.51855 | **0.048828** | 0.42383 | |
| p-value | 0.2766 | | 0.12125 | | 0.12125 | | 0.81748 | | 0.10621 | | 0.092681 | | 0.080575 | | |
| **Fz** |  |  |  |  |  |  |  |  |  |  |  |  |  |  | |
| Mean (SD) | -7.1122 (14.7172) | 4.5255 (22.2303) | 3.657 (8.3789) | -0.87911 (9.0426) | 4.041 (11.677) | -3.0781 (14.9066) | 0.21247 (2.1179) | -0.53773 (2.3635) | -0.18124 (0.32407) | -0.042999 (0.58337) | -0.29364 (0.62791) | -0.016491 (0.67757) | -0.3154 (0.38854) | 0.029504 (0.42175) | |
| Median | -3.4132 | -0.70259 | 2.1681 | 1.0789 | 2.1123 | -1.6066 | 0.0013869 | -0.067315 | -0.16565 | 0.073749 | -0.19968 | 0.10006 | -0.24566 | 0.053969 | |
| Min, Max | -39.3944, 7.2725 | -21.5594, 49.3208 | -10.9909, 19.7251 | -26.2861, 10.6419 | -9.8309, 30.7999 | -40.2357, 22.7269 | -2.3168, 3.7793 | -6.2663, 2.2362 | -0.63359, 0.29824 | -1.644, 0.56018 | -1.3153, 0.76401 | -1.4484, 1.0794 | -0.97617, 0.16335 | -0.84266, 0.70295 | |
| p-value | 0.23242 | 0.96973 | 0.23242 | 0.56934 | 0.375 | 0.62207 | 0.8457 | 0.7334 | 0.16016 | 0.79102 | 0.19336 | 0.7334 | **0.027344** | 0.67725 | |
| p-value | 0.44828 | | 0.40981 | | 0.24853 | | 0.66822 | | 0.19852 | | 0.24853 | | 0.051754 | | |
| **F1** |  |  |  |  |  |  |  |  |  |  |  |  |  |  | |
| Mean (SD) | -8.7305 (15.5111) | 6.6135 (24.0239) | 5.8392 (10.1228) | -3.4996 (8.9826) | 3.4634 (10.952) | -2.7054 (14.5001) | 0.67156 (2.8465) | -0.86382 (3.6016) | -0.2371 (0.36959) | 0.0022911 (0.67564) | -0.53423 (0.58562) | 0.20625 (0.76257) | -0.46118 (0.49971) | 0.24333 (0.61611) | |
| Median | -1.3195 | 7.3949 | 4.2235 | -2.174 | 0.025792 | -3.8912 | -0.29263 | -0.63675 | -0.17914 | 0.090152 | -0.42505 | 0.31634 | -0.33262 | 0.1198 | |
| Min, Max | -37.5427, 4.6041 | -23.4642, 49.3004 | -5.8293, 30.6454 | -26.2982, 6.3982 | -6.7678, 29.9922 | -34.3037, 25.0992 | -3.1197, 6.1783 | -6.2471, 4.9278 | -0.72736, 0.38191 | -1.6383, 0.97269 | -1.2228, 0.43512 | -1.4422, 1.2915 | -1.1118, 0.26444 | -0.55333, 1.6226 | |
|  | 0.375 | 0.51855 | 0.10547 | 0.33936 | 0.8457 | 0.38037 | 0.625 | 0.42383 | 0.083984 | 0.79102 | **0.019531** | 0.33936 | **0.019531** | 0.17627 | |
|  | **Delta** | | **Theta** | | **Alpha** | | **Beta** | | **High-beta** | | **Gamma** | | **High-gamma** | | |
|  | **Intervention  Group (N=10)** | **Control Group (N=12)** | **Intervention  Group (N=10)** | **Control Group (N=12)** | **Intervention  Group (N=10)** | **Control Group (N=12)** | **Intervention  Group (N=10)** | **Control Group (N=12)** | **Intervention  Group (N=10)** | **Control Group (N=12)** | **Intervention  Group (N=10)** | **Control Group (N=12)** | **Intervention  Group (N=10)** | **Control Group (N=12)** | |
| p-value | 0.375 | 0.51855 | 0.10547 | 0.33936 | 0.8457 | 0.38037 | 0.625 | 0.42383 | 0.083984 | 0.79102 | 0.019531 | 0.33936 | 0.019531 | 0.17627 | |
| p-value | 0.092681 | | 0.044314 | | 0.17646 | | 0.40981 | | 0.13791 | | 0.032114 | | 0.013411 | | |
| **F3** |  |  |  |  |  |  |  |  |  |  |  |  |  |  | |
| Mean (SD) | -6.1669 (15.5857) | 4.7375 (23.5104) | 3.9187 (4.9142) | -2.404 (8.7572) | 3.0318 (12.3448) | -2.4995 (14.192) | -0.043567 (2.815) | -0.57494 (3.2121) | -0.1958 (0.83301) | 0.07106 (0.74553) | -0.30466 (1.5145) | 0.30415 (0.97159) | -0.23469 (1.2828) | 0.35792 (0.78782) | |
| Median | -2.3313 | 1.8443 | 3.331 | 0.96155 | 2.4716 | -2.4947 | -0.20949 | 0.60665 | -0.159 | 0.28578 | -0.45281 | 0.27287 | -0.44227 | 0.16443 | |
| Min, Max | -38.4175, 12.8467 | -22.0528, 51.0156 | -1.5295, 13.0727 | -26.3504, 4.7977 | -19.3307, 30.3534 | -35.8447, 24.9404 | -4.1931, 4.0601 | -6.3933, 3.8285 | -1.4343, 1.7438 | -1.6754, 1.0473 | -2.4218, 3.4937 | -1.5323, 1.9977 | -1.8488, 3.0662 | -0.65051, 1.6641 | |
| p-value | 0.32227 | 1 | 0.064453 | 0.56934 | 0.32227 | 0.51855 | 1 | 0.7334 | 0.19336 | 0.62207 | 0.10547 | 0.26611 | 0.10547 | 0.26611 | |
| p-value | 0.48872 | | 0.092681 | | 0.19852 | | 0.71686 | | 0.17646 | | 0.069786 | | 0.037797 | | |
| **F5** |  |  |  |  |  |  |  |  |  |  |  |  |  |  | |
| Mean (SD) | -6.1741 (12.1006) | 4.6613 (22.8538) | 5.2822 (6.1494) | -3.1826 (8.3774) | 4.1854 (9.4894) | -2.9227 (13.8173) | -0.776 (2.5453) | -0.55462 (3.1806) | -0.458 (0.93763) | 0.3819 (1.2416) | -1.0422 (1.2446) | 0.89098 (2.1313) | -0.97722 (1.0325) | 0.69818 (1.5469) | |
| Median | -5.7431 | 0.085462 | 4.8222 | -0.7976 | 4.5933 | -2.0381 | -0.98645 | 0.50887 | -0.25565 | 0.4103 | -0.75995 | 0.64649 | -0.63351 | 0.39021 | |
| Min, Max | -30.4105, 12.2936 | -29.4573, 50.1464 | -1.7541, 13.6973 | -26.3099, 4.181 | -13.0283, 21.1631 | -31.2364, 28.1912 | -3.8809, 3.2378 | -6.4624, 3.6895 | -2.3232, 0.85089 | -1.6961, 3.4907 | -4.0309, 0.16496 | -1.5589, 6.8589 | -2.6886, 0.11863 | -1.1495, 4.5002 | |
| p-value | 0.16016 | 0.7334 | 0.083984 | 0.26611 | 0.23242 | 0.15137 | 0.27539 | 0.96973 | 0.16016 | 0.33936 | **0.0097656** | 0.15137 | **0.0097656** | 0.17627 | |
| p-value | 0.15629 | | 0.022914 | | 0.092681 | | 0.66822 | | 0.10621 | | 0.011129 | | 0.0091994 | | |
| **F7** |  |  |  |  |  |  |  |  |  |  |  |  |  |  | |
| Mean (SD) | -9.1913 (10.0989) | 6.0511 (21.4797) | 4.628 (8.3135) | -2.5662 (8.2817) | 2.6903 (5.8687) | -3.8533 (11.5502) | 0.29329 (1.9108) | -0.65222 (2.8269) | 0.43681 (1.0026) | 0.13977 (0.70506) | 0.76741 (1.6949) | 0.40597 (1.0543) | 0.37962 (1.2927) | 0.44958 (1.1198) | |
| Median | -12.7607 | -0.25943 | 2.8214 | -0.29796 | 1.207 | -1.106 | 0.40678 | 0.10297 | -4.5704e-05 | 0.24698 | 0.19582 | 0.41341 | 0.077721 | 0.33253 | |
| Min, Max | -18.9735, 14.5029 | -19.3623, 50.1259 | -8.0966, 19.834 | -26.3137, 4.2355 | -3.5174, 12.1059 | -30.0082, 15.8295 | -3.0293, 3.8414 | -6.458, 4.0384 | -0.70047, 2.1579 | -1.6924, 0.84232 | -0.86174, 4.698 | -1.5544, 2.2353 | -1.0563, 2.7142 | -0.78644, 3.3237 | |
|  | **Delta** | | **Theta** | | **Alpha** | | **Beta** | | **High-beta** | | **Gamma** | | **High-gamma** | | |
|  | **Intervention  Group (N=10)** | **Control Group (N=12)** | **Intervention  Group (N=10)** | **Control Group (N=12)** | **Intervention  Group (N=10)** | **Control Group (N=12)** | **Intervention  Group (N=10)** | **Control Group (N=12)** | **Intervention  Group (N=10)** | **Control Group (N=12)** | **Intervention  Group (N=10)** | **Control Group (N=12)** | **Intervention  Group (N=10)** | | **Control Group (N=12)** |
| p-value | **0.027344** | 0.79102 | 0.16016 | 0.38037 | 0.27539 | 0.2334 | 0.76953 | 0.62207 | 0.55664 | 0.2334 | 0.19336 | 0.20361 | 0.625 | 0.26611 | |
| p-value | 0.044314 | | 0.092681 | | 0.24853 | | 0.53105 | | 1 | | 0.9737 | | 0.62093 | | |
| **FC1** |  |  |  |  |  |  |  |  |  |  |  |  |  |  | |
| Mean (SD) | -4.856 (18.7243) | 3.8414 (24.5761) | 3.4201 (11.1088) | -1.0724 (8.9335) | 2.2874 (11.5971) | -2.4634 (15.1969) | -0.0042233 (2.3015) | -0.5847 (3.2681) | -0.24362 (0.38599) | 0.028819 (0.66664) | -0.33472 (0.56756) | 0.1074 (0.70028) | -0.26013 (0.46024) | 0.14009 (0.42136) | |
| Median | -2.8258 | -0.40688 | 0.18273 | 1.1194 | 2.4946 | -0.68505 | -0.9083 | -0.064702 | -0.34856 | 0.26466 | -0.35392 | 0.23548 | -0.27114 | 0.084213 | |
| Min, Max | -32.2878, 29.0171 | -25.1247, 55.4168 | -9.6037, 30.497 | -26.2797, 7.6305 | -22.304, 19.5254 | -41.4838, 21.0901 | -3.1677, 4.1632 | -6.2818, 5.1364 | -0.67093, 0.56961 | -1.6381, 1.0089 | -1.1309, 0.89209 | -1.4506, 1.0788 | -1.0679, 0.72647 | -0.52399, 0.9518 | |
| p-value | 0.49219 | 0.96973 | 0.625 | 0.67725 | 0.43164 | 0.79102 | 0.69531 | 0.62207 | 0.083984 | 0.42383 | **0.048828** | 0.33936 | 0.064453 | 0.2334 | |
| p-value | 0.57516 | | 0.86907 | | 0.48872 | | 0.81748 | | 0.092681 | | 0.051754 | | 0.02718 | | |
| **FC3** |  |  |  |  |  |  |  |  |  |  |  |  |  |  | |
| Mean (SD) | -1.7724 (14.4776) | 3.6882 (24.1462) | 2.971 (7.1039) | -1.6121 (8.7273) | 1.8702 (12.9454) | -1.6382 (15.0822) | -1.257 (2.181) | -0.55308 (3.4859) | -0.44799 (0.56733) | -0.0091901 (0.71243) | -0.58985 (0.61502) | 0.05943 (0.81368) | -0.74217 (1.1654) | 0.064246 (0.62671) | |
| Median | -0.64835 | -2.2217 | 2.1026 | -0.23682 | -0.45457 | 0.53425 | -1.5615 | 0.1379 | -0.28474 | 0.040232 | -0.5126 | 0.084482 | -0.39721 | 0.047293 | |
| Min, Max | -37.5976, 10.9464 | -23.7745, 52.5466 | -9.6277, 13.1913 | -26.2346, 6.5999 | -21.0384, 29.4364 | -37.6695, 26.3933 | -4.5716, 2.5036 | -6.6685, 5.0496 | -1.8749, 0.092175 | -1.6666, 0.99902 | -1.8236, 0.10327 | -1.5009, 1.0798 | -3.8068, 0.11939 | -1.0251, 1.0631 | |
| p-value | 0.92188 | 0.62207 | 0.27539 | 0.90967 | 0.8457 | 0.8501 | 0.13086 | 0.8501 | **0.0058594** | 0.79102 | **0.019531** | 0.79102 | **0.013672** | 0.7334 | |
| p-value | 0.81748 | | 0.40981 | | 0.71686 | | 0.48872 | | 0.080575 | | **0.044314** | | 0.060213 | | |

**Supplementary Table 17. Relative band power difference (Week 4 – Baseline) of parietal EEG**

|  | **Delta** | | **Theta** | | **Alpha** | | **Beta** | | **High-beta** | | **Gamma** | | **High-gamma** | |
| --- | --- | --- | --- | --- | --- | --- | --- | --- | --- | --- | --- | --- | --- | --- |
|  | **Intervention  Group (N=10)** | **Control Group (N=12)** | **Intervention  Group (N=10)** | **Control Group (N=12)** | **Intervention  Group (N=10)** | **Control Group (N=12)** | **Intervention  Group (N=10)** | **Control Group (N=12)** | **Intervention  Group (N=10)** | **Control Group (N=12)** | **Intervention  Group (N=10)** | **Control Group (N=12)** | **Intervention  Group (N=10)** | **Control Group (N=12)** |
| **CP3** |  |  |  |  |  |  |  |  |  |  |  |  |  |  |
| Mean (SD) | 0.96105 (15.727) | 6.9187 (24.7503) | 2.1173 (8.6117) | -3.2439 (8.1567) | -1.5527 (9.326) | -2.8763 (17.3135) | -0.65718 (1.9955) | -1.2315 (3.2512) | -0.31281 (0.4064) | -0.0076716 (0.82469) | -0.28876 (0.58502) | 0.16792 (1.3251) | -0.25971 (0.45531) | 0.26208 (0.94641) |
| Median | 5.6865 | 0.90888 | 1.5597 | -0.030406 | -2.5117 | -2.4813 | -0.92231 | -0.63767 | -0.25059 | 0.1502 | -0.17283 | 0.1456 | -0.20777 | 0.19307 |
| Min, Max | -33.9202, 17.1282 | -32.2421, 51.915 | -15.5471, 13.2296 | -26.4394, 3.6853 | -13.6015, 19.1199 | -34.6185, 35.6482 | -3.2481, 2.4994 | -7.1218, 3.1031 | -1.0697, 0.22045 | -1.6583, 1.0534 | -1.5037, 0.36747 | -2.3802, 2.3252 | -1.021, 0.34635 | -1.6316, 1.9326 |
| p-value | 0.625 | 0.56934 | 0.49219 | 0.42383 | 0.27539 | 0.46973 | 0.375 | 0.33936 | **0.037109** | 0.90967 | 0.16016 | 0.51855 | 0.13086 | 0.26611 |
| p-value | 0.9737 | | 0.24853 | | 0.92121 | | 0.92121 | | 0.19852 | | 0.24853 | | 0.080575 | |
| **P1** |  |  |  |  |  |  |  |  |  |  |  |  |  |  |
| Mean (SD) | 0.0705 (25.914) | 0.99384 (28.8087) | -3.3278 (9.3359) | -2.4771 (10.3784) | 7.9867 (22.7824) | 0.7547 (16.8051) | -2.2326 (3.4558) | 0.20583 (3.728) | -0.62889 (1.3601) | 0.071951 (0.73536) | -0.99302 (2.7571) | 0.25729 (0.91883) | -0.84961 (2.7309) | 0.18929 (0.4975) |
| Median | -2.7575 | 1.3792 | -2.7242 | 0.70852 | 6.6955 | -1.492 | -1.6361 | 0.33786 | -0.29803 | 0.15012 | -0.31449 | 0.25789 | -0.15961 | 0.14911 |
| Min, Max | -36.4609, 51.4773 | -42.6984, 50.5426 | -21.2409, 13.5154 | -25.9413, 11.2146 | -24.5195, 53.3773 | -30.0095, 25.2791 | -10.6947, 1.754 | -6.5045, 6.1369 | -4.4224, 0.24228 | -1.648, 1.1744 | -8.6148, 1.3056 | -1.4423, 2.3245 | -8.383, 1.5315 | -0.5075, 1.4636 |
| p-value | 0.92188 | 0.90967 | 0.27539 | 0.79102 | 0.55664 | 1 | **0.027344** | 0.79102 | **0.048828** | 0.51855 | 0.23242 | 0.26611 | 0.27539 | 0.26611 |
| p-value | 0.9737 | | 0.53105 | | 0.62093 | | 0.15629 | | 0.060213 | | 0.10621 | | 0.15629 | |
| **P3** |  |  |  |  |  |  |  |  |  |  |  |  |  |  |
| Mean (SD) | 0.1943 (31.1039) | 4.1893 (29.8167) | -1.8936 (12.2757) | -1.9913 (9.6788) | 4.8935 (18.647) | -2.268 (19.1329) | -1.9715 (3.0346) | -0.28245 (3.6904) | -0.46393 (0.61046) | 0.01718 (0.79823) | -0.45074 (0.50338) | 0.12903 (1.2542) | -0.29924 (0.3487) | 0.19965 (0.73215) |
| Median | 3.1001 | 6.4637 | -1.3347 | -1.4782 | 1.0328 | -2.8903 | -2.0836 | -0.48612 | -0.36207 | -0.015399 | -0.34895 | 0.05164 | -0.20105 | 0.088637 |
| Min, Max | -57.5471, 45.1298 | -42.5759, 52.5584 | -21.7074, 18.1078 | -26.1524, 11.659 | -20.093, 47.417 | -36.9147, 38.0933 | -8.2155, 2.138 | -6.7903, 5.5867 | -2.0941, 0.07579 | -1.638, 1.3732 | -1.5665, 0.020551 | -2.1857, 2.7925 | -0.84802, 0.066369 | -1.0424, 1.8187 |
| p-value | 1 | 0.42383 | 0.49219 | 0.62207 | 0.8457 | 0.42383 | 0.064453 | 0.67725 | **0.0058594** | 1 | **0.0097656** | 0.67725 | **0.019531** | 0.46973 |
|  | **Delta** | | **Theta** | | **Alpha** | | **Beta** | | **High-beta** | | **Gamma** | | **High-gamma** | |
|  | **Intervention  Group (N=10)** | **Control Group (N=12)** | **Intervention  Group (N=10)** | **Control Group (N=12)** | **Intervention  Group (N=10)** | **Control Group (N=12)** | **Intervention  Group (N=10)** | **Control Group (N=12)** | **Intervention  Group (N=10)** | **Control Group (N=12)** | **Intervention  Group (N=10)** | **Control Group (N=12)** | **Intervention  Group (N=10)** | **Control Group (N=12)** |
| p-value | 0.53105 | | 0.9737 | | 0.37338 | | 0.24853 | | 0.092681 | | 0.12125 | | 0.069786 | |
| **P5** |  |  |  |  |  |  |  |  |  |  |  |  |  |  |
| Mean (SD) | -3.9114 (21.9565) | 2.7483 (32.5282) | 1.9594 (9.9347) | -2.5278 (9.7994) | 3.4483 (12.8185) | -0.94717 (22.6902) | -0.71083 (1.1214) | 0.16074 (3.6351) | -0.23981 (0.1957) | 0.14948 (0.99003) | -0.303 (0.37412) | 0.15857 (1.9008) | -0.23624 (0.50545) | 0.24362 (1.3527) |
| Median | 0.56304 | 2.6844 | 1.3554 | -2.305 | 0.48138 | -2.919 | -1.0063 | 1.4617 | -0.18403 | 0.26595 | -0.1906 | 0.19636 | -0.11041 | 0.23301 |
| Min, Max | -46.3244, 39.2295 | -65.3837, 58.2171 | -19.6933, 19.8255 | -26.2426, 12.7122 | -17.6357, 28.1985 | -44.9365, 48.8698 | -1.9392, 1.4713 | -6.7602, 4.6143 | -0.62558, -0.050944 | -1.5779, 2.0015 | -1.0394, 0.10605 | -4.2826, 4.025 | -1.4409, 0.29914 | -2.9166, 3.139 |
| p-value | 0.76953 | 0.96973 | 0.43164 | 0.42383 | 0.55664 | 0.79102 | 0.10547 | 0.79102 | **0.0019531** | 0.38037 | **0.013672** | 0.42383 | 0.16016 | 0.17627 |
| p-value | 0.62093 | | 0.22252 | | 0.44828 | | 0.24853 | | **0.037797** | | **0.037797** | | 0.080575 | |
| **POZ** |  |  |  |  |  |  |  |  |  |  |  |  |  |  |
| Mean (SD) | -1.4935 (28.4127) | 9.157 (24.4595) | 1.6724 (5.4523) | -3.5693 (8.1983) | 2.4607 (25.4841) | -4.5971 (16.698) | -1.0793 (1.8401) | -0.63386 (2.9393) | -0.36917 (0.52024) | -0.15921 (0.60323) | -0.60129 (0.81413) | -0.11544 (0.71839) | -0.57171 (0.8128) | -0.077623 (0.49769) |
| Median | -0.71939 | 6.2626 | 0.51158 | -1.0754 | 2.0942 | -2.1061 | -0.74613 | -0.69777 | -0.19432 | -0.12398 | -0.17168 | -0.10778 | -0.10859 | -0.062572 |
| Min, Max | -45.7386, 46.9087 | -24.298, 60.9067 | -5.2536, 10.8565 | -26.0873, 3.6544 | -44.4798, 40.3998 | -50.989, 15.2725 | -4.8641, 1.2489 | -6.5313, 5.462 | -1.6058, 0.077647 | -1.693, 0.88836 | -2.391, 0.072771 | -1.4733, 1.4352 | -2.1387, 0.20242 | -0.86682, 1.1503 |
| p-value | 0.92188 | 0.2334 | 0.43164 | 0.26611 | 0.625 | 0.42383 | 0.083984 | 0.30127 | **0.019531** | 0.26611 | **0.013672** | 0.38037 | 0.064453 | 0.42383 |
| p-value | 0.33902 | | 0.15629 | | 0.37338 | | 0.86907 | | 0.57516 | | 0.37338 | | 0.33902 | |
| **P2** |  |  |  |  |  |  |  |  |  |  |  |  |  |  |
| Mean (SD) | 4.8927 (22.0507) | 9.7152 (24.5121) | 0.69853 (5.5517) | -5.0215 (8.5852) | -3.3922 (18.8301) | -6.6255 (11.6055) | -1.0731 (2.7145) | -0.23781 (4.197) | -0.30106 (0.51337) | 0.32495 (1.6384) | -0.44648 (0.69816) | 0.93256 (3.2653) | -0.36606 (0.65771) | 0.88292 (2.9604) |
| Median | -0.055445 | 6.9645 | 0.33185 | -5.1222 | -1.0053 | -4.7975 | 0.16287 | -0.5474 | -0.0010212 | -0.041087 | -0.082375 | -0.023461 | -0.083644 | -0.026513 |
| Min, Max | -29.3749, 37.84 | -30.9397, 53.3192 | -9.6717, 11.5766 | -26.078, 4.1695 | -35.5527, 23.4097 | -37.4486, 11.8575 | -6.7808, 1.4327 | -6.5211, 8.9459 | -1.4414, 0.087582 | -1.6917, 4.9951 | -1.5246, 0.14716 | -1.4745, 10.82 | -1.5971, 0.21299 | -0.79344, 10.0237 |
|  | **Delta** | | **Theta** | | **Alpha** | | **Beta** | | **High-beta** | | **Gamma** | | **High-gamma** | |
|  | **Intervention  Group (N=10)** | **Control Group (N=12)** | **Intervention  Group (N=10)** | **Control Group (N=12)** | **Intervention  Group (N=10)** | **Control Group (N=12)** | **Intervention  Group (N=10)** | **Control Group (N=12)** | **Intervention  Group (N=10)** | **Control Group (N=12)** | **Intervention  Group (N=10)** | **Control Group (N=12)** | **Intervention  Group (N=10)** | **Control Group (N=12)** |
| p-value | 0.69531 | 0.15137 | 0.55664 | 0.052246 | 0.23242 | **0.026855** | 0.55664 | 0.56934 | 0.43164 | 0.96973 | 0.19336 | 0.90967 | 0.23242 | 0.96973 |
| p-value | 0.40981 | | 0.15629 | | 0.19852 | | 0.9737 | | 0.57516 | | 0.40981 | | 0.48872 | |
| **P4** |  |  |  |  |  |  |  |  |  |  |  |  |  |  |
| Mean (SD) | 5.7451 (25.145) | 3.5643 (29.0997) | 3.457 (8.412) | -1.686 (9.7649) | -6.8691 (20.0638) | -4.2704 (21.3298) | -0.9892 (2.9315) | 0.15449 (4.1136) | -0.40867 (0.71915) | 0.37461 (1.5467) | -0.48983 (0.61921) | 0.92411 (3.1487) | -0.4322 (0.62578) | 0.9084 (2.8782) |
| Median | 0.79478 | 0.77017 | 1.3707 | -0.28775 | -6.0201e-05 | -2.6418 | -0.2135 | 0.327 | -0.18656 | 0.15877 | -0.28572 | 0.096761 | -0.11875 | 0.12208 |
| Min, Max | -28.7437, 56.7184 | -35.3125, 61.9834 | -5.5971, 20.0953 | -25.746, 12.732 | -44.9893, 16.3951 | -49.9689, 40.7214 | -7.1902, 2.6793 | -6.5618, 6.5919 | -2.2983, 0.10533 | -1.6841, 4.7173 | -1.6015, 0.12348 | -1.4786, 10.4604 | -1.7672, 0.11561 | -0.87857, 9.8049 |
| p-value | 0.625 | 0.90967 | 0.55664 | 0.67725 | 0.55664 | 0.26611 | 0.49219 | 0.90967 | 0.13086 | 0.56934 | **0.019531** | 0.62207 | **0.027344** | 0.51855 |
| p-value | 0.76668 | | 0.40981 | | 0.81748 | | 0.62093 | | 0.13791 | | 0.13791 | | 0.10621 | |
