# Supplementary material for: Double-blind, sham-controlled, pilot study of trigeminal nerve stimulation for autism spectrum disorder
Source: Neurotherapeutics. 2026 Jan 29;23(1):e00838. doi: 10.1016/j.neurot.2026.e00838 (PMC12976484; doi:10.1016/j.neurot.2026.e00838)
Supplement: Multimedia component 2 [file mmc2.pdf]

Protocol

A single-center, double-blind, placebo-controlled, randomization, exploratory clinical trial to evaluate the safety and efficacy of NuEyne P01 (stimulator for personal medical use) in mitigation of symptoms related to autism spectrum disorder in patients with autism spectrum disorder

Protocol No.	NE_PSY_001
Protocol Version	3.0
Date of Finalization of Protocol	2024.02.02
Product Name	Electric stimulator for personal medical use (Grade 3)

Protocol Synopsis

Clinical Trial Title	A single-center, double-blind, placebo-controlled, randomization, exploratory clinical trial to evaluate the safety and efficacy of NuEyne P01 (stimulator for personal medical use) in mitigation of symptoms related to autism spectrum disorder in patients with autism spectrum disorder
Clinical Trial Purpose	The purpose of this trial is to evaluate the safety and efficacy of NuEyne P01 (stimulator for personal medical use) in relation to symptoms of autism spectrum disorder, including social interaction, autistic features and social anxiety, in patients with autism spectrum disorder after applying a trigeminal nerve stimulation using the device to the experimental group and control group.
Trial Design	Single-center, double-blind, placebo-controlled, randomization, exploratory clinical trial
Trial Subject	A patient with autism spectrum disorder
Sponsor	Nu Eyne, Co., Ltd.
Investigational Device	Electric stimulator for personal medical use (Grade 3)
Clinical Trial Institution	Seoul National University Bundang Hospital
Principal Investigator	Professor Hee Jeong YOO
Clinical Trial Period	About 12 months from the date of approval by the Ministry of Food and Drug Safety
Target Number of Subjects	30 in total (Drop-out rate 20% considered)
Inclusion/Exclusion Criteria	**Inclusion Criteria**<br>1. A child of age not less than 7 and less than 12<br>2. A patient diagnosed with autism spectrum disorder<br>**(The diagnosis is finally determined based on the ADOS-2 (autism diagnostic observation schedule version 2) and K-ADI-R (autism diagnostic interview-revised) indices and the principal investigator's clinical decision.)**<br>3. An individual with the overall intelligence score not less than 70 who has no problem with linguistic communication

	**(For the intelligence score, a subject with the K-WISC-IV index not less than 70 is included.)**<br>4. An individual who agrees to not using other investigational devices than the investigational device during the clinical trial period<br>5. An individual who will not use any allowed concomitant drugs until the end of the clinical trial or who is capable of maintaining the currently used allowed concomitant drug without any changes or additions during the clinical trial period<br>*** Allowed Concomitant Drug: Psychostimulant (methylphenidate), norepinephrine reuptake inhibitor (atomoxetine), antipsychotics (aripiprazole, risperidone etc.), selective serotonin reuptake inhibitor (escitalopram, sertraline, fluoxetine, paroxetine etc.)**<br>6. An individual who voluntarily provides consent to participation in the clinical trial<br>(Or an individual whose legally authorized representative provides the consent)<br><br>**Exclusion Criteria**<br>1. An individual who participated in another clinical trial within 30 days from the screening<br>2. An individual with a limited intelligence score or linguistic ability as to have a difficulty understanding the treatment and performing the trial activities<br>3. An individual with a clinically significant behavioral problem, emotional regulation problem, psychosis symptoms, or risks of causing an injury to oneself or others as to affect the treatment course<br>4. An individual with a history of psychiatric hospitalization<br>5. An individual with acute or chronic severe medical psychiatric disease<br>6. An individual with a medical history of seizure<br>7. An individual who had a major trauma or surgery within 1 month<br>8. An individual who is considered to have a problem with attachment of electrodes of the investigational device due to inflammatory reaction in the forehead skin or other dermatological problems<br>9. An individual who is considered to fall under other reasons for contraindications of the investigational device (e.g. cautions and contraindications of the product, including heart-related problems, seizure, a metal or electronic implant in the head, unexplained pain,

	pacemaker implant)<br>* The investigational device has little correlation with other in vitro devices because it causes local stimulation to the head<br>10. An individual who needs to use drugs of a hypnotic class (the benzodiazepine class and melatonin and trazodone)<br>(However, the individual may enter the clinical trial after having a washout period of at least 2 weeks.)<br>11. An individual who is not cooperative with the treatment and is considered not suitable for continuous voluntary participation in the trial in the investigator's opinion<br>12. An individual who is otherwise considered inappropriate for participation in the trial in the investigator's opinion
Trial Methodology	A written consent is obtained from a patient who satisfies all of the inclusion criteria without falling under any of the exclusion criteria. Up to 4 outpatient visits occur during the clinical trial period. The screening visit, start day visit, and Visits occurring at Weeks 2 and 4 after the start day together with tests applicable to each visit will occur.<br>① Subject informed consent<br>② Eligibility check based on the inclusion/exclusion criteria<br>③ Subject enrollment<br>④ Investigational device application (or Sham stimulation device)<br>- **Experimental group (15 subjects):** The electric stimulator for personal medical use is applied to the forehead and surrounding area for 28 days, **7 times/week, 8 hours (±60 minutes)/application every day**, starting from the start day. During the trial period, the existing drug treatments and tests used for autism spectrum disorder will continue the same but use of any other investigational devices is prohibited. **(Concomitant drug/therapy is allowed)**<br>- **Control group (15 subjects):** An investigational device having the same appearance as the investigational device with no actual functioning effect is applied to the forehead and surrounding area for 28 days, **7 times/week, 8 hours (±60 minutes)/application every day**, starting from the start day. During the trial period, the existing drug treatments and tests used for autism spectrum disorder will continue the same but use of any other investigational devices is prohibited.<br>**(Concomitant drug/therapy is allowed)**

⑤ Safety and efficacy evaluations

Investigational Device

- Name and Manufacturer
  - Item name: Electric stimulator for personal medical use (Grade 3)
  - Item classification no.: A83000
  - Grade: 3
  - Model name: NuEyne P01
  - Packaging unit: 1 set
  - Manufacturing site: Nu Eyne, Co., Ltd., 608, 6F, 28, Digital-ro 30-gil, Guro-gu, Seoul, Republic of Korea
- Appearance and Structure

1) SMILE main body (control no. TPD-P01)

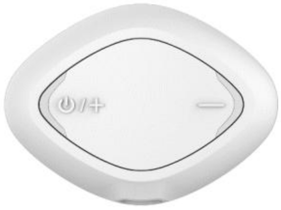 Front	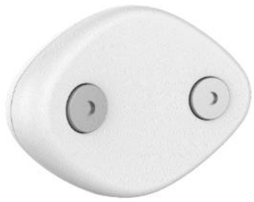 Back

2) Electrode (product report no. 19-1321) (PS-2HP2)

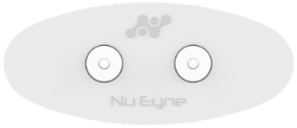 Front	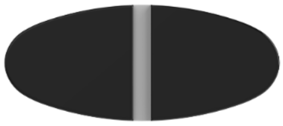 Back

3) Device wearing

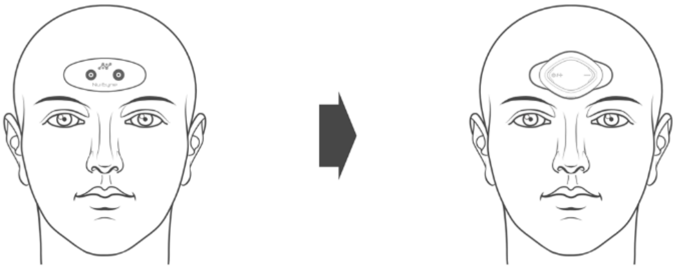

- Performance

Clinical trial maximum output time: 8 hours

Clinical trial output current: 2 to 4 mA

Clinical trial maximum output voltage: 2 V ±1 V (Load=500 Ω)

	Clinical trial frequency: 120Hz<br>Clinical trial pulse phase duration: 250 μs<br>Clinical trial session<br>- 30 sec ON / 1 sec lamping down / 30 sec OFF / 1 sec lamping up
Endpoint	Efficacy endpoint<br>1. Primary endpoint: Safety evaluation<br>The safety of pulse electric stimulation of 4 weeks is compared to the baseline.<br>Occurrence of adverse events caused by the investigational device, including psychiatric abnormality, skin abnormality at the contact site, headache, sleepiness, trigeminal nerve abnormality, etc. is checked.<br><br>2. Secondary endpoint: Efficacy evaluation<br>Evaluation of the level of mitigation of autism spectrum disorder based on the mean difference in the following efficacy endpoints<br>within each group and between the groups:<br>- Change to the level of functioning at Week 4 compared to the baseline<br>- Change to the social reciprocity at Weeks 2 and 4 compared to the baseline<br>- Change to the executive function at Weeks 2 and 4 compared to the baseline<br>- Change to the sleep disturbance at Weeks 2 and 4 compared to the baseline<br>- Change to the anxiety at Weeks 2 and 4 compared to the baseline<br>- Change to the sensory over – responsivity at Weeks 2 and 4 compared to the baseline<br>- Change to the clinical global impression at Weeks 2 and 4 compared to the baseline
Expected effect and result	● Expected effect<br>The transcutaneous stimulation will act on the brain stem and cerebrum areas involved in various cognitive functions and emotions, including locus coeruleus, reticular activating system and nucleus tractus solitarius, and will have an effect of mitigating autism spectrum disorder symptoms.<br>● Expected result<br>After application of NuEyne P01, there will be a significant difference the efficacy endpoints, including sleep condition, anxiety symptoms, sensory sensitivity, attention and core symptoms of autism, compared to before application.

Clinical Trial Flow Diagram

Observation Item		Visit 1 (Screening)	Visit 2	Visit 3 (Tele-visit 1)	Visit 4	Visit 5 (Tele-visit 2)	Visit 6 (EOT)
		-4 weeks	0 day (Baseline)	1 weeks (±3 days)	2 weeks (±3 days)	3 weeks (±3 days)	4 weeks (+3 days)
Trial explanation and informed consent		●					
Eligibility check based on the inclusion/exclusion criteria		●	●				
Vital sign check		●	●		●		●
Demographics and development history check		●					
Concomitant drug check		●	●	●	●	●	●
Physical examination		●	●		●		●
Autism spectrum diagnosis (K-ADOS2, K-ADI-Revised)		●					
Laboratory test	General blood test	●					●
	General chemistry test	●					●
12-lead ECG		●					●
Brainwave test	Quantitative EEG	●					●
	Waking EEG						
Subject enrollment			●				
Efficacy evaluation 10)	Level of functioning		●				●
	Social reciprocity		●		●		●
	Executive function		●		●		●
	Sleep disturbance		●		●		●
	Anxiety		●		●		●
	Sensory over – responsivity		●		●		●
	Clinical Global impression (CGI)		●				●
Investigational device and app use training			●	(●)	(●)	(●)	
Investigational device and diary distribution			●				
Investigational device application			●	●	●	●	
Investigational device compliance check				●	●	●	●
Device usability questionnaire							●
Adverse event check		●	●	●	●	●	●
Investigational device collection							●
